# Wild and zoo-housed orangutans differ in how they explore objects

**DOI:** 10.1038/s41598-025-97926-z

**Published:** 2025-04-30

**Authors:** Isabelle B. Laumer, Shubhangi Kansal, Anais van Cauwenberghe, Tri Rahmaeti, Tatang Mitra Setia, Roger Mundry, Daniel Haun, Caroline Schuppli

**Affiliations:** 1 Development and Evolution of Cognition Research Group, Max Planck Institute of Animal Behavior, Konstanz, Germany; 2 University of Leipzig, Leipzig, Germany; 3 Department of Biology, Graduate Program, Faculty of Biology and Agriculture, Universitas Nasional, Jakarta, 12520 Indonesia; 4 Fakultas Biologi, Universitas Nasional, Jakarta, Indonesia; 5 Department for Primate Cognition, Georg-August-Universität Göttingen, Johann-Friedrich-Blumenbach Institute, Kellnerweg 4, 37077 Göttingen, Germany; 6 Cognitive Ethology Laboratory, German Primate Center, Leibniz Institute for Primate Research, Kellnerweg 4, 37077 Göttingen, Germany; 7 Leibniz Science Campus Primate Cognition, 37077 Göttingen, Germany; 8 Department of Comparative Cultural Psychology, Max Planck Institute for Evolutionary Biology, Leipzig, Germany; 9 Department of Evolutionary Anthropology, University of Zurich, Zurich, Switzerland

**Keywords:** Exploration, Exploratory object manipulation, Captive-wild comparison, Cognitive development, Cognitive aging, Great ape, Developmental biology, Evolution

## Abstract

In human infants, exploratory object manipulations (henceforth called “EOM”) stimulate cognitive development and affect cognitive performance in later life. Zoo-housed great apes are frequently used to study the evolution of human cognition, however, it is unknown how the zoo environment affects their daily expression of EOM. We investigated how wild and zoo-housed Sumatran orangutans differ in their daily EOM throughout life. We collected ~ 12′000 EOM events by 51 wild and zoo-housed individuals of all ages. Zoo-housed orangutans showed significantly higher EOM rates than wild orangutans. Exploratory actions were more diverse in zoos than in the wild, even with objects available in both settings. Zoo-housed orangutans also showed a larger repertoire of exploratory actions and a higher probability of multi-object exploration, including tool use. There was no difference between settings at which age individuals first showed specific exploratory actions. Our results show that the zoo environment significantly affects EOM in orangutans and that the species’ exploratory potential exceeds its natural expression. This may have important implications for cognitive performance, as zoo-housed individuals are likely to have a broader range of affordances to draw from when confronted with novel problems. These results highlight the potential of captive-wild comparisons to study cognitive development and evolution.

## Introduction

Exploratory object manipulation (i.e., the deliberate manipulation and visual inspection of objects^1–5^, henceforth referred to as “EOM”) enables human infants to learn about objects and their physical properties (e.g. texture, weight, gravity, etc.^1,3,4,6–10^). EOM is also an important vehicle of cognitive stimulation^11^ as it is linked to motor and cognitive development^12–20^. Neurological studies further provide evidence for a link between EOM and cognitive processing^21–23^. In human infants, manual, visual, and oral EOM peaks around the age of two years and then decreases^3,24–26^. However, the length of EOM events increases^3,20,27,28^, and EOM becomes more diverse and complex with increasing age (e.g., a larger number and different exploratory actions are combined;^1,3,24,28^). These patterns are indicative of learning processes as actions become more fine-tuned and more goal-directed over time^29^.

Similar to humans, EOM also leads to the development of knowledge and sensorimotor skills in non-human animals (e.g.^30–35^). Studies in various mammalian and bird species have shown that EOM correlates positively with problem-solving ability and innovation probability^36–43^. This suggests that knowledge and experience gained during EOM can be transferred across contexts and applied to novel problems. Thus, similar to humans, EOM also appears to be a key mechanism in non-human animals for translating cognitive potential into actual knowledge and skills as well as for fostering cognitive performance in several domains (e.g.,^5,44^).

In primates, EOM tendency is closely linked with phylogeny in that strepsirrhine primates show the lowest levels of EOM tendency, followed by Platyrrhines, non-hominid Catarrhines, and great apes^45^. Non-human great apes (henceforth called “great apes”) show the most diverse and complex EOMs of all non-human primates in terms of rates of bimanual manipulation, object-object (i.e., two or more are combined), and object-substrate manipulations (i.e., an object is actively combined with a substrate, other than the plain floor) as well as the use of different body parts during manipulation^3,46–49^. Humans demonstrate even more complex and diverse manipulations^50,51^. Despite these differences, manipulations present across multiple taxa develop in a highly similar sequence, suggesting relatively conserved neurodevelopmental processes^52,53^. Furthermore, like humans, wild great apes show a peak in exploratory tendency during the early dependency period^5,33^ and an increase of EOM diversity with increasing age^5^.

In large-brained species with pronounced developmental plasticity, cognition is shaped by the social and ecological environment^54,55^. In humans, the conditions under which one grows up influence cognitive development (e.g.,^56–58^), and cognitive performance is shaped by specific experiences during development^59^. Children growing up with severely limited social and physical stimulation later show deficits in several cognitive domains, along with physiological and anatomical changes in the brain (e.g.,^57,60^). Similarly, rhesus monkeys (*Macaca mulatta*) that grow up in socially deprived conditions perform worse in various cognitive tasks and have significantly altered brain structures (^61^, see^62^ for similar findings in rats). Contrarily, high frequency and quality of social inputs during early childhood positively affect cognitive development in humans^63^. Accordingly, enculturation (i.e., being raised with close human contact) positively affects performance in physical and social cognition tasks in great apes^64–69^. Effects of the ecological and social environment on the development of cognition appear to be, at least to some degree, mediated through exploration behavior, including EOM ^42,68,70^.

Zoo-housed great apes are frequently used as a model for studying human cognition. Compared to their wild conspecifics, they are easier to access and observe, and can be tested under controlled conditions. This allows for minimizing confounding effects of the social and ecological environment during testing. In contrast, performing tests with wild great apes is challenging for a variety of factors, such as their high levels of neophobia ^71,72^ and high protection status due to associated risks such as disease transmission from humans to the test subjects (e.g., ^73^). As cognitive studies on zoo-housed great apes often draw conclusions on the evolutionary level, it is crucial to increase our understanding of how the zoo environment affects their cognitive performance. Wild and zoo-housed individuals are exposed to different physical and social environments, which result in differences in the cognitive demands they face^71–77^. On the one hand, wild great apes face cognitive challenges that are reduced or absent in captivity, such as the need to locate food sources in the habitat, defend their territories, or hide from predators^56^. On the other hand, zoo-housed great apes grow up in close contact with humans and thus likely receive more varied social stimulation^68^. Furthermore, zoo-housed great apes are provided with carefully designed, at times even computer-assisted enrichment and thus objects that vary in their properties (e.g., shape, size, color, material) and can be manipulated in myriads of ways (e.g.^76,75–80^). Zoo-housed great apes thus likely gain a broad range of affordances from EOM, that they may transfer to novel situations (i.e., when they manipulate a novel object or are confronted with novel problems, e.g.^81^).

Zoo-housed individuals also have increased amounts of time and energy available for exploration because their food is regularly provided and they do not have to travel to find food or mates. So far, it has remained largely unclear how these factors affect great apes’ cognitive performance.

Using EOM as a measure of cognitive performance may help to quantify cognitive differences in wild and captive great apes. Even though the developmental trajectories for the development of the overall frequency of EOM are to some degree hard-wired^5,33^, there is evidence that captivity increases overall exploration tendency in great apes (e.g.,^71,82,83^). However, so far, detailed comparisons of the development of EOM in wild versus zoo-housed great apes across ages are lacking. Because individuals can experience cognitive stimulation through EOM throughout the day, to be able to quantify potential differences in cognitive stimulation through EOM, one needs to compare exploration during their daily lives in their habitat (henceforward called “daily EOM behavior”). Because the effects of captivity on cognition may change throughout an individual’s life, it is important to study cognitive differences across a large age range. To date, systematic comparisons of wild and captive animals’ daily EOM behavior and comparisons across large age ranges are missing.

Orangutans (*Pongo sp.*) are an ideal study species for investigating the effect of the environment on exploration, as their high tendencies to explore allow for studying the details of the behavior (e.g.^5,30^). Furthermore, whilst the mainly arboreal lifestyle of wild orangutans (e.g.^84^) requires them to hold onto branches and thus likely limits exploration, zoo-housed orangutans spend more time on the ground (e.g.^85^) and thus often have both hands free for exploration. Furthermore, wild orangutans are challenging to study because of their low numbers and resulting high protection status, and because their habitats present challenges to human observers. It is therefore crucial to understand whether zoo-housed orangutans differ significantly from wild orangutans. To adress this, we compared different aspects of daily EOM behavior in wild and zoo-housed orangutans across all ages and predicted that:

P1a) The age trajectories of the overall frequency of EOM are similar in zoo-housed and wild orangutans.

P1b) Zoo-housed orangutans of all ages show more frequent EOM and longer EOM bouts than wild orangutans.

P2) Zoo-housed orangutans of all ages exhibit a greater diversity in their EOM in that they a) use more exploratory actions, even when exploring the same objects, b) use more body parts to explore, and c) are more likely to explore multiple objects.

P3) The ages at which individuals first show each behavior will be similar across the two settings.

In our study, we defined EOM as the prolonged, non-repetitive, usually destructive manipulations of objects (with or without a tool), excluding feeding and nest building, while the visual and tactile foci (manual and/or oral) of the individual are on the object. We analysed a balanced data set containing 12′000 EOM events of wild and zoo-housed Sumatran orangutans. Because most EOM happens during the immature period (below the age of 16)^5^ and to prevent overall trends across all ages from overwriting effects during the immature period, we present separate analyses on the immature period in the supplementary information.

## Results

### 1a) Developmental trajectory of EOM frequency

**Fig. 1:**
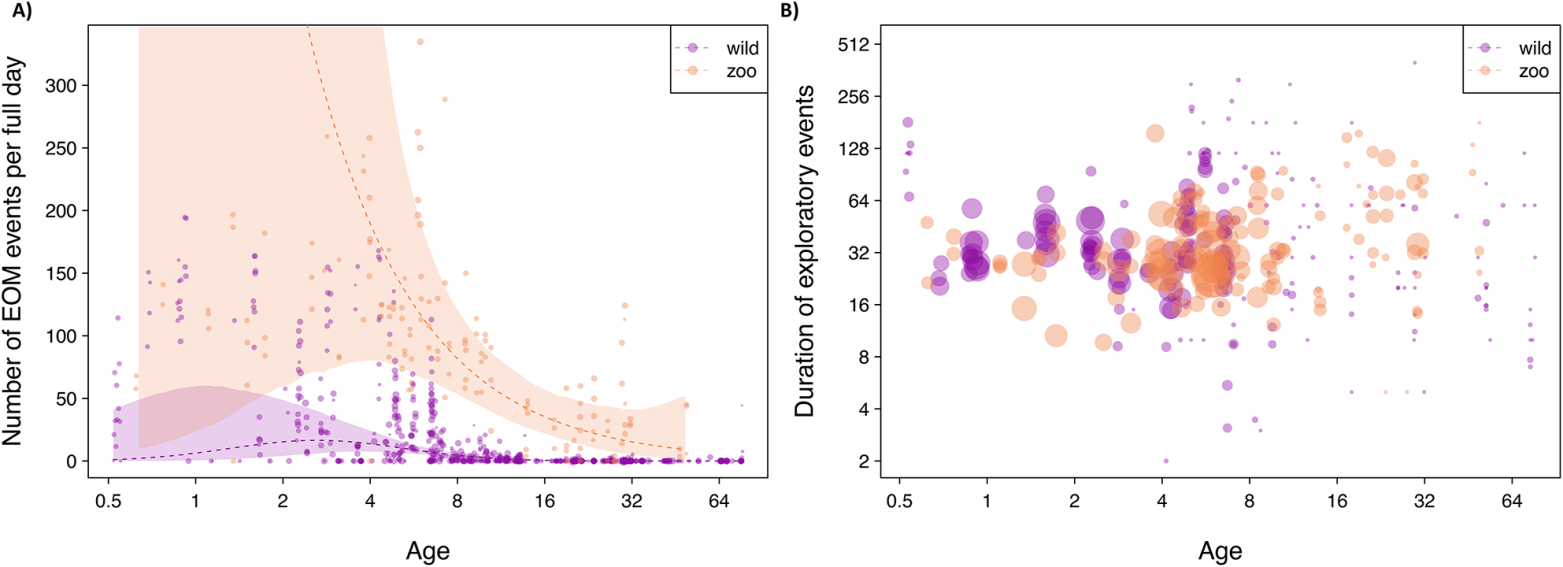
(**a**) EOM frequency. Number of EOM events per day (i.e., 12 h; as this roughly corresponds to the wake time of a wild orangutan^86^) as a function of age at both settings (wild: purple; zoo-housed: yellow). The points depict the EOM rate per a given age, per setting, and their area corresponds to the total duration of observations (range of actual observation durations per dot: 0.08 to 22.21 h). The dashed lines and shaded areas depict the fitted model and its 95% confidence limits. (**b**) EOM duration. Duration of EOM events as a function of age in wild (purple) and zoo-housed orangutans (yellow). The points depict the average duration of EOM events per age and setting and their area corresponds to the number of observations (range: 1 to 275).

Age-dependent EOM rates significantly differed between wild and zoo-housed orangutans (full-null model comparison, likelihood ratio test (LRT): χ2 = 114.362, df = 4, P < 0.001), and there was a significant interaction between setting and age squared (χ2 = 7.410, df = 1, P = 0.006), indicating setting-specific age effects; SI, section D1, Table S5). Inspecting the data and the fitted model revealed that zoo-housed individuals generally explored more frequently than wild individuals. Furthermore, in wild orangutans, EOM rates peaked at around two to three years, whereas in zoo-housed orangutans, statistically no such peak was detected (Fig. 1a). However, notably, the fitted model was associated with large uncertainly (wide confidence intervals in Fig. 1a) and revealed large fitted values for 0.5 to 2-year-old zoo animals.

### 1b) Duration of EOM

The age-dependent EOM duration per EOM event was not significantly different between settings as indicated by a non-significant full-null model comparison (LRT: χ2 = 4.839, df = 3, P = 0.184, see Fig. 1b and SI, D, Table S6 for more details; median _zoo_: 30 s [10 s, 80 s], median _wild_: 30 s [15 s, 60 s]). See SI, D, 1b.2 for the results of the models on EOM duration in immature individuals only, where also no significant difference between the settings was found.

### 2a) EOM diversity: actions

**Fig. 2:**
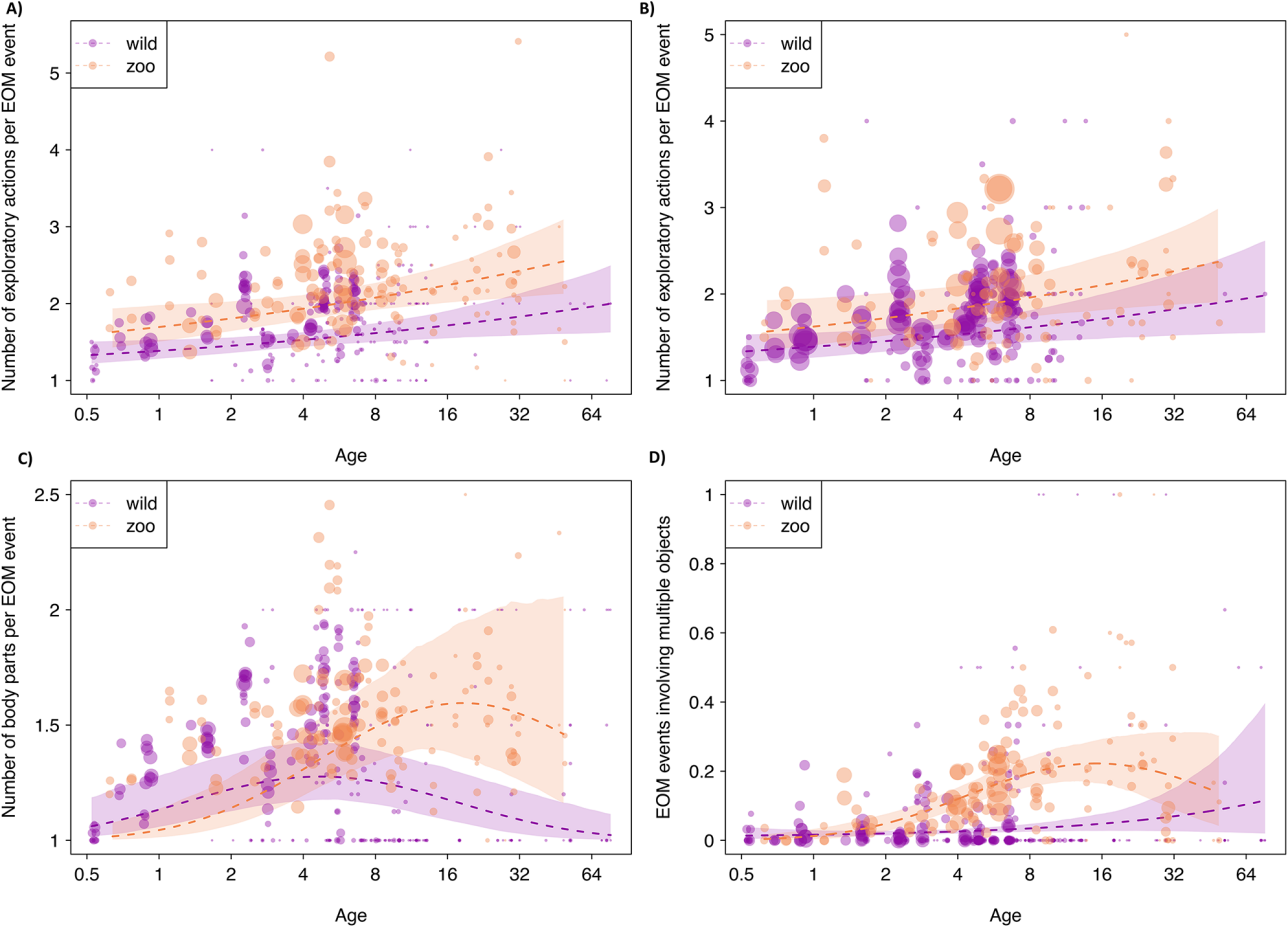
Exploration action diversity for all EOM events (**a**), exploration action diversity for all events involving only natural objects (**b**), exploration body part diversity (**c**), and probability to involve multiple objects in explorations (**d**) as a function of age in wild (purple) and zoo-housed orangutans (yellow). Dots show the average response per age and setting, whereby the area of the dots corresponds to the number exploration events per dot (ranges: a: 1 to 273; b: 1 to 70, c: 1 to 276; d: 1 to 280). Dashed lines and shaded areas in all plots depict the fitted model and its 95% confidence limits.

When looking at exploration events including all object types, the age-dependent number of exploratory actions performed per EOM event differed significantly between wild and zoo-housed orangutans (LRT: χ2 = 17.019, df = 3, P < 0.001). However, the interaction between setting and age squared was not significant in this model (χ2 = 1.251, df = 1, P = 0.263; SI, section D2a, Table S8). After the removal of this non-significant interaction, we also did not find a significant effect of age squared (χ2 = 1.525, df = 1, P = 0.217; SI, section D2a, Table S9) which indicated linear age trajectories and led us to remove age squared from the model. In the resulting model, also the interaction between age and setting was not significant (χ2 = 0.049, df = 1, P = 0.825; SI, section D2a, Table S10) and we thus removed it from the model. The final model revealed significant effects of setting (χ2 = 15.620, df = 1, P < 0.001; SI, section C3a, Table S11) and age (χ2 = 7.140, df = 1, P = 0.008; SI, section D2a, Table S11). The number of exploratory actions per EOM event increased with age in a similar fashion in wild and zoo-housed orangutans, but zoo-housed individuals performed significantly more exploratory actions per event (Fig. 2a; median number of actions _zoo_ = 2 [1, 3], median number of actions _wild_ = 2 [1, 2]). The results of the models on exploration action diversity in immature individuals only showed a significant difference in the same direction between the settings (SI, section D, 2a.2).

### 2b) EOM action diversity: EOM events involving objects available in both settings

When looking at exploration events including natural objects that occur at both settings, the age-dependent number of exploratory actions performed per EOM event was significantly different between zoo-housed and wild orangutans (LRT: χ2 = 10.000, df = 3, P = 0.019; see SI, D, S16). After the iterative removal all non-significant effects involving interactions and age squared (see SI, D, Table S16-S18) we found significant effects of setting (χ2 = 8.462, df = 1, P = 0.004; see SI, D, Table S19) and age (χ2 = 5.299, df = 1, P = 0.021; see SI, D, Table S19) in that zoo-housed orangutans used more exploratory actions when exploring objects that are available at both settings (see Fig. 2b). Accordingly, the average number of exploratory actions was higher in zoo-housed than in wild orangutans (median number of actions _zoo_ = 2 [1, 3], median number of actions _wild_ = 2 [1, 2]). See SI, D, 2b.2 for the results of the models on exploration action diversity in immature individuals only, where a significant difference between the settings in the same direction was found.

### 2c) EOM diversity: body parts

The age-dependent trajectory of the body parts used per EOM event was significantly different between wild and zoo-housed orangutans (LRT: χ2 = 11.935, df = 3, P = 0.008; SI, section D2c, Table S24). The interaction between age squared and setting was not significant in this model (χ2 = 0.042, df = 1, P = 0.838). After the removal of this interaction, the final model revealed a significant interaction between age and setting (χ2 = 8.600, df = 1, P = 0.003; SI, D2c, Table S24) and a significant effect of age squared (χ2 = 11.304, df = 1, P = 0.001; SI, D2c, Table S25). Whilst in wild individuals the number of body parts used to explore peaked at the age of four to eight, in zoo-housed orangutans it peaked at about 16 years of age (see Fig. 2c; median number of body parts _zoo_: 1 [1, 2]; median number of body parts _wild_: 1 [1, 2]). See SI, D2c. 2 for the results of the models on exploration body part diversity in immature individuals only, where a significant difference in the same direction between the settings was found.

### 2d) EOM diversity: objects

There was a significant difference between the settings in the age-dependent probability that EOM events involved more than one object (LRT: χ2 = 47.983, df = 3, P < 0.001). The interaction between age squared and setting was significant in this model (χ2 = 5.924, df = 1, P = 0.015); for the model results see SI, section D4.1, Table S28). In zoo-housed orangutans, the probability that EOM events involved multiple objects increased until the age of 16 and then decreased, while overall the probability of EOM events involving multiple objects was higher in zoo-housed compared to wild orangutans (see Fig. 2d). Wild orangutans showed an overall low probability to involve multiple objects in their exploration throughout all ages. Among the immature individuals, we found a significant difference in the same direction between the settings for the probability that EOM events involved more than one object (SI, D4.2).

### 3) Age of first occurrence of specific EOM

**Fig. 3:**
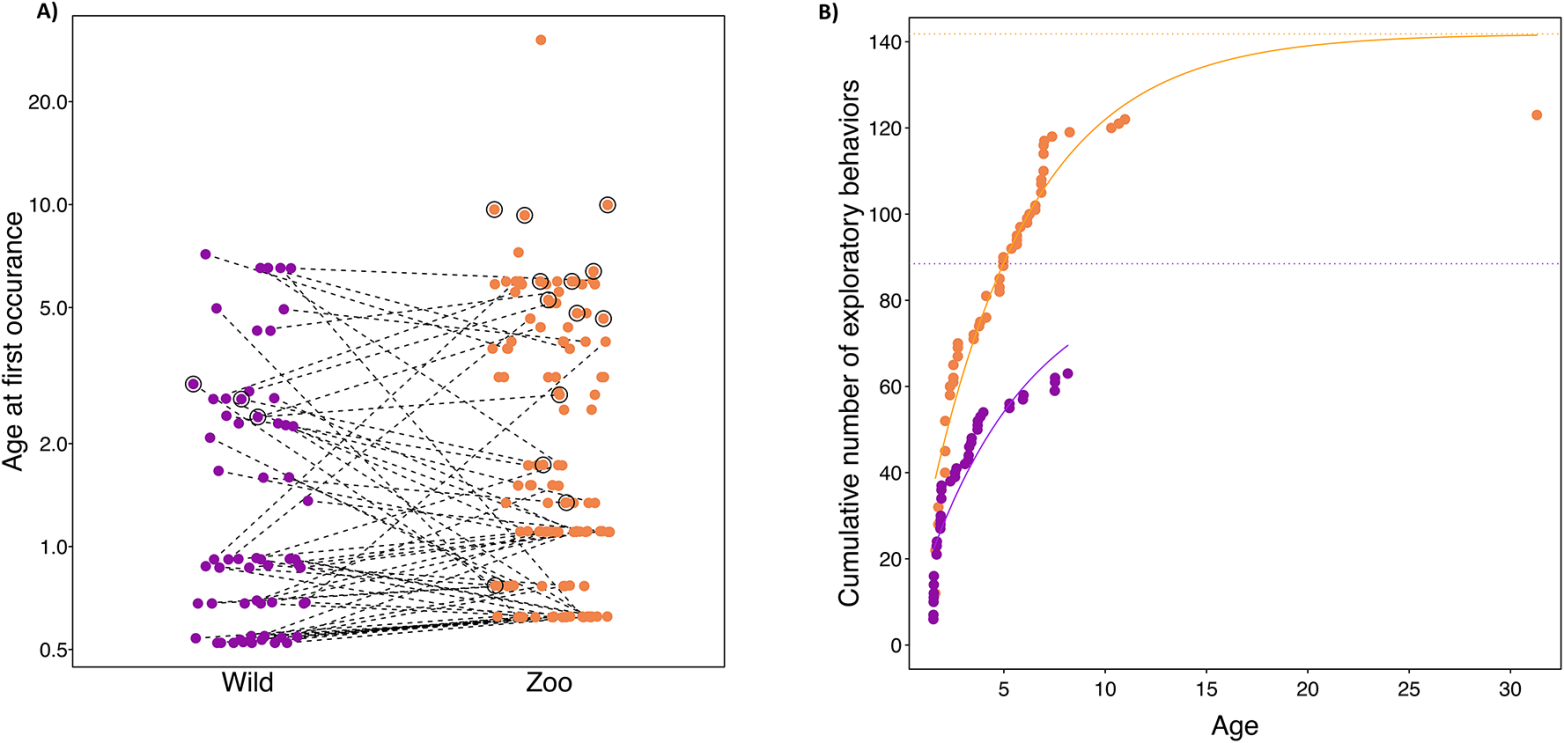
(**a**) Age at first occurrence of exploratory actions. Age of first occurrence of all exploratory actions in wild and zoo-housed orangutans. Note that the corresponding analysis (see above) only included actions that were performed by individuals in both settings. Dots surrounded by circles indicate exploratory actions that include the use of a tool. Dashed lines connect the same respective exploratory action. **b) **Age trajectories for repertoires of exploratory actions. Cumulative number of exploratory actions as a function of age in wild (purple) and zoo-housed orangutans (yellow). Solid lines depict the fitted model and dashed lines indicate the respective asymptote, i.e., the estimated total repertoire size at adult age.

We did not find a significant difference between settings in the age at which individuals first showed exploratory actions that occurred at both settings (paired-samples t-test: t (60) = –0.8112, p = 0.421; see Fig. 3a). However, overall zoo-housed orangutans showed a larger repertoire of EOMs (n = 123) than wild orangutans (n = 63; for a detailed list see SI, section D3a, Table S32). Additionally, we also found a larger number of tool-orientated EOM in zoo-housed individuals compared to wild individuals (n_zoo_ = 13 vs. n_wild_ = 3, Table S32, Fig. 3a (indicated by circles around the respective dots)).

Almost all EOM were observed for the first time below the age of ten years in both settings (see Fig. 3b). The cumulative number of EOM as a function of age in zoo orangutans showed a higher asymptote (estimate = 0.472, SE = 0.129, z = 3.654, p < 0.001; see Fig. 3b and SI, section D3b, Table S33) but the slope of the increase did not differ significantly between settings (estimate = -0.044, SE = 0.210, z = -0.210, p = 0.834; see Fig. 3b and SI, section D3b, Table S33). However, note that cumulative repertoire sizes were determined pooled across all individuals for the analysis, and therefore do not allow for conclusions on the level of individuals’ repertoire development.

## Discussion

To assess potential effects of the zoo environment on cognition, we investigated differences in EOM between zoo-housed and wild orangutans across a wide age range. Taken together, we found overall similar age-dependent patterns of daily EOM rates for zoo-housed and wild orangutans. However, compared to wild orangutans, zoo-housed immature orangutans showed the highest EOM rates later in development and continued to show higher EOM rates throughout late immaturity and adulthood . We did not find a difference in individuals’ EOM duration between the two settings. Zoo-housed orangutans used a greater variety of exploratory actions and body parts to explore. Furthermore, zoo-housed orangutans showed more tool-orientated behaviors and a higher probability of EOM involving multiple objects. Despite larger overall repertoires of exploratory actions observed in zoo-housed orangutans, there was no difference between settings in the age at which different exploratory actions were first shown.

Consistent with findings in human and non-human primates^3,5,24–26,33^, we found overall similar age trajectories of EOM rates (P1a) characterized by a steep increase during the early dependency period, followed by a decrease in both settings. This similarity across the vastly different settings suggests that the age-dependent motivation to engage in EOM is not affected by immediate socio-ecological conditions nor the conditions experienced during development, indicating that it may have hard-wired components. Furthermore, in both settings, EOM became more diverse with increasing age (P2), which is indicative of learnin^29^. This pattern suggests that just as human infants, wild and zoo-housed immature orangutans learn about objects through EOM.

As predicted (P1b), zoo-housed orangutans showed more frequent EOM at all ages in their daily lives which is in line with previous studies in captive great apes (e.g.,^68,71^). Living in safe and stable captive conditions, animals may experience increased free time and energy to devote to EOM while not being occupied with time-intensive foraging or vigilance (e.g.,^82,87^). Wild great apes have been found to be less curious than zoo-housed great apes^71^, which is commonly attributed to differences in levels of neophobia^88^. Whilst studies on curiosity commonly measure individuals’ reactions to novel objects, our study analyzed EOM performed with the orangutans’ naturally encountered objects and standard enrichment (the enrichment scheme mostly entails objects that are provided regularly; see SI, table S1b). The difference in EOM tendencies of familiar objects between the settings cannot be caused by differences in neophobia and instead likely reflects a generally heightened exploratory tendency in zoo-housed orangutans, which may also partly underlie the observed differences in curiosity between wild and captive individuals. Overall, our findings on higher exploration rates in zoo-housed orangtuans are in line with a study on wild orangutans’ curiosity, which showed that visual exploration of novel objects increases with increasing food availability^89^.

The difference in EOM rates between the settings was less pronounced for immatures below four years of age, most likely because they are still suckling, which provides energetic buffering and reduces the impact of foraging demands on their activity budgets. Interestingly, at around the age of eight years, when wild Sumatran orangutans are typically weaned and a younger sibling is born (for Suaq, the average weaning age is 8.1 years, Schuppli unpublished data), their EOM rates dropped to near zero values. In contrast, zoo-housed immatures showed a peak in EOM rates at this age, despite much shorter interbirth intervals of approximately 5.5 years^90,91^ and thus earlier weaning. The fact that the drop in EOM frequency coincides with weaning in the wild but not in the zoos supports the theory that exploration is affected by individuals’ need to forage for themselves. In contrast to our prediction (second part of prediction P1b), EOM durations did not differ between settings, indicating that, even though wild orangutans overall showed lower rates of EOM, *when* exploring, orangutans at both settings were equally persistent in doing so.

As we predicted, zoo-housed orangutans showed more diverse EOM (in terms of number of actions performed and body parts used to explore; P2) than their wild counterparts. In undisturbed wild habitats (such as the Suaq research area), orangutans encounter a stable array of natural objects, ranging from tree substrate (e.g., bark, leaves, etc.) to epiphytes, fungi, as well as bird nests. While these objects can be inherently complex and their configurations vary, they only allow for certain exploratory behaviors. Modern zoos, including the zoos where we collected our data, provide specifically designed enrichment consisting of objects of different shapes and materials which likely allows for a large range of exploratory actions (e.g.,^76,75–80^). Interestingly, when exploring natural objects that occur at both settings, zoo-housed individuals also showed a greater diversity of exploratory actions (second part of prediction P2). The latter result implies that the difference in EOM diversity across settings is more than a direct result of differences in the objects that are being explored. Instead, this suggests that individuals of the two settings differ in the set of affordances (i.e., the range of actions) that they can draw from when exploring, most likely because they transfer their repertoires of actions across contexts, i.e., explored objects.

Ultimately, these differences in affordances may also affect cognitive performance in other domains, such as problem-solving. Indeed, studies show that zoo-housed animals often show better task performance in problem-solving experiments than their wild counterparts (^70^, but see e.g.^92^), a phenomenon known as the ‘captivity bias’ (^87,93^. This is also reflected by higher rates of innovative behavior in captivity compared to wild settings^94–97^. However, aside from being the result of opportunities to explore different objects, differences in cognitive performance between wild and zoo-housed individuals may also be caused by social factors. Studies have shown that great apes that have experienced extensive interactions with humans exhibit enhanced cognitive performance in the physical^64,98,99^ and social domain (e.g.^67^ but see^100^). An experiment with captive and rehabilitated orangutans with different rearing backgrounds found that the degree of human orientation predicted the motivation to explore an apparatus which affected their problem-solving skills^68^, suggesting that the effects of the social and physical environment on cognitive performance interact with each other. To pin down in what aspects and to what extent differences in daily EOM behavior translate into differences in cognitive performance, systematic standardized cognitive tests in both settings are needed which are to date lacking for great apes (but see, e.g.,^70,101^ for findings on other species).

Zoo-housed orangutans also showed a higher probability of exploring multiple objects in one event (P2). Zoo enrichment likely allows for more complex and sustained combinatory object-object and object-substrate manipulations than naturally occurring objects^76,75–80^. Combinatory object manipulations seem to be linked to abilities in the domain of physical cognition, as tool-using species show the most complex structured object combinations (e.g. corvids and parrots^44^, primates^49^). Combinatory actions have also been shown to be relevant to the development of tool-use abilities in humans^102^. Even though we found evidence for more multi-object manipulations in zoo-housed orangutans, we could not investigate the exact nature of these manipulations because our data only entail information on how many distinct objects were explored (but not how many objects of the same type). Therefore, we do not know whether the actions performed were combinatory or not. However, our results showed that zoo-housed orangutans have a larger repertoire of tool-orientated behaviors than wild orangutans. The greater availability of objects that allow for more complex, including more combinatory manipulations, may stimulate cognitive development^71–77^.

Most exploratory actions were first shown by individuals below the age of ten years in both settings. Furthermore, there was no difference between the settings in the ages of first occurrence for exploratory actions that were present at both settings (P3), which supports the conclusion of previous studies that the neurodevelopmental sequence of manipulative skills is largely fixed in primates (see^52,53^). However, zoo-housed orangutans showed an overall higher number of exploratory actions than wild orangutans (P2) which could be a result of having different object types available to explore.

Interestingly, several of our findings showed that certain aspects of EOM change throughout life, indicating that the developmental effects on EOM are not restricted to the immature period. In both settings, body part diversity during exploration decreased after a certain age whereas action diversity increased with age. This may suggest that orangutans become more skilled as they age and therefore can perform a more diverse range of actions while using a smaller number of body parts. If EOM is directly linked to cognitive performance, our results suggest that cognitive performance, similarly as in humans (e.g.,^103,104^), in great apes also changes throughout adulthood.

## Conclusion

Taken together, our results show that the zoo environment significantly affects EOM in orangutans as well as that aspects of EOM and the effect of captivity on EOM change throughout life. Our findings also strengthen existing evidence that EOM reflects cognitive development and might function as a means of cognitive stimulation, not just in humans but across great apes.

The comparison across settings suggests that the conditions that individuals experience at the zoo amplify their daily EOM behavior. Given the link between EOM and certain areas of cognitive performance (e.g. problem-solving and innovation probability^36–43^), our findings contribute to growing evidence that, in some aspects, cognitive performance measured in zoo-housed individuals may not be directly comparable to that of their wild counterparts^64–67^. On the one hand, this calls for caution when drawing conclusions on the evolutionary level, such as on the evolution of cognition. This is especially true for studies that compare cognitive performance across species by testing captive individuals because the strength of the effect of captivity on EOM and cognitive performance may vary across different species. On the other hand, differences in EOM and cognitive performance between captive and wild great apes allow for important novel insights into the proximate factors affecting cognition which are in themselves informative about the evolution of cognition. Accordingly, captive studies may help to elucidate a species’ cognitive potential, i.e., the level of cognitive abilities individuals can develop upon the evolved cognitive potential they are born with.

## Methods

### Subjects

The data on wild Sumatran orangutans were collected from 2007 to 2020 at the Suaq Balimbing research area located in the Gunung Leuser National Park in South Aceh, Indonesia (3°42’N, 97°26’E). The data on zoo-housed Sumatran orangutans were collected from 2021 to 2023 at Leipzig Zoo and Dresden Zoo in Germany, and Zoo Zurich and Basel Zoo in Switzerland (for information on enclosure size and enrichment schedule see SI, Table S1b). All-occurrence event data (that were used for models 2–4; see below) included 6478 EOM events performed by 33 wild orangutans on 363 observation days between the ages of 0.5 and 76.7 years at Suaq Balimbing in Indonesia and 6640 EOM events performed by 24 zoo-housed orangutans on 134 observation days between the ages of 0.6 and 49.4. Note that sample sizes varied for the different analyses, as not all details were reported for all events (for subject information see SI, section A, Table S1a). Note that a subset of the EOM data in wild immature orangutans (3200 EOM events collected on 13 immatures between the ages of 0.5 and 13 years) has already been published previously^5^. Following van Noordwijk et al. 2018, we call individuals below the age of 16 “immatures”, below the age of 8 years “dependent immatures” (at Suaq the average weaning age is 8.1 years; Schuppli, unpublished data), and between the age of 8 and 16 years “independent immatures”. Individuals above the age of 16 are referred to as “adults”.

### Ethical guidelines

The data collection was purely observational and without any interaction with the study animals. The research protocols for the data collection in the wild were approved by the Ministry of research and technology (RISTEK; research permit no. 152/ SIP/FRP/SM/V/2012 and following permits) and complied with the legal requirements of Indonesia. Data collection at the zoos did not lead to any deviation from the usual husbandry protocols, was approved by the ethics joint committee of the Max Planck Institute for Evolutionary Anthropology, Leipzig Zoo, and was carried out in accordance with relevant guidelines and regulations at Leipzig Zoo, Dresden Zoo, Basel Zoo, and Zoo Zurich. Our research protocols complied with the EAZA minimum standards for the accommodation and care of animals in zoos and aquaria. The research adhered to all German and Swiss laws regarding animal keeping.

### Data collection procedure

The data were collected following an established protocol for orangutan behavioral data collection(ab.mpg.de/571,325/standarddatacollectionrules_suaq_detailed_jan204.pdf ) which consisted of a) instantaneous scan sampling at two-minute intervals of the individual’s activity and b) all-occurrence sampling of behaviors of interest, including EOM. We collected the data on wild orangutans during focal animal follows, of which 50.9% were full-day follows (i.e., from when the focal animal got out of its nest in the morning until it went into its evening nest) and 49.1% partial follows (when the focal animal was either found and then followed until the evening nest or followed from the morning nest until it was lost). The zoo data were collected from the moment the orangutans entered their enclosure in the morning until they left it in the evening (or from the beginning until the end of the visitor hours, in case the orangutans remained in the enclosure for 24 h). The zoo-housed orangutans were observed from the visitors’ area or an observation tower (at Leipzig Zoo), which provides a view of the indoor enclosure from above. EOM was defined as the prolonged, non-repetitive, usually destructive manipulations of objects (with or without a tool), while the visual and tactile foci (manual and/or oral) of the individual are on the object^105^ (see movie S1 for an example of EOM in the wild and in zoo-housed orangutans). Following Pisula^106^, we excluded feeding with or without a tool (defined as such by actual ingestion), but included object play (i.e., manipulations during which the visual and tactile foci were desynchronized, and were characterized by repetitive movements), as it is at times difficult to reliably distinguish exploratory from non-exploratory object play (but see^107,108^). Further we did not include nesting behavior. The exception was if the orangutan was deconstructing a nest or was manipulating twigs that may have been unsuccessful attempts to start constructing a nest. This was done as it is hard to judge the individual’s intention in these instances. We coded the duration of EOM events from the start of the manipulation to the time the orangutan stopped manipulating. To be counted as two separate EOM events, there had to be at least ten seconds between two EOM events during which no EOM was observed.Data were collected by 25 experienced observers (see SI, section A2 for details on inter-observer reliability).

### Data preparation

To investigate EOM frequencies, we counted the number of EOM events per observation day (model 1a). We included data from 65 wild and 24 zoo-housed orangutans (collected on 650 and 142 observation days respectively, note that we also included days where the EOM rate was zero). To investigate EOM duration (model 1b), we used the duration of all EOM events that were timed. To investigate exploratory action diversity (model 2a), we used the number of different exploratory actions performed during EOM events (SI, section B, Table S2a for a list of all explorative actions and definitions). We also assessed exploratory action diversity for EOM events that included natural objects only that occurred in both settings (SI, section B, Table S2b; model 2b). To investigate the diversity of body parts involved during EOM (model 2c), we used the number of body parts involved (see SI, section B, Table S3 for a list of all body parts). To assess EOM object diversity (model 2d), we coded whether or not multiple distinct objects (i.e., two or more) were manipulated during an event (see SI, Table S2b).

### Statistical analysis

To assess how the age-dependent development of various aspects of EOM differed between settings, we fitted general and Generalized Linear Mixed Models (LMM and GLMM;^109^) using a significance criterium of P < 0.05. In all models, we included setting and age as fixed effects predictors. To account for the possibility that EOM rates peaked at intermediate ages, we also included age squared into the fixed effects part of each model. As the age-dependent trajectory of EOM rate could vary between settings, we also included the interaction between age and setting and age squared and setting in the fixed effects part of all models. We further included random intercepts effect of the individual ID, and in models 1b – 2d we included follow number (i.e., the id of the observation day) nested in subject as a further random intercepts effect (as in the all-occurrence data, events associated with individuals other than the focal of the follow were recorded which resulted in data on multiple individuals under the same follow number). When identifiable, to account for individual variation in age trajectories and to avoid overconfident models and increased type I error rates, we also included random slopes^110,111^ of age and age squared.

To assess the overall effects of the predictors, we first compared each full model with a respective null model which lacked setting and its interactions with age and age squared in the fixed effects part (also in the zero inflation part in model 1a) but was otherwise identical to the full model via a likelihood ratio test (LRT,^112^). In case a full-null model comparison revealed significance, we first determined the significance of the interaction between setting and age squared, and if this did not reveal significance, we removed it from the model and then checked the significance of the effect age squared. If this also did not reveal significance, we removed it from the model, too, and checked the significance of the interaction between age and setting. In case this interaction did not reveal significance, we also removed it from the model (see^113^). This iterative removal of non-significant interactions served in obtaining unconditional estimates of the respective lower-order terms^114^.

#### EOM frequency and duration

To investigate differences in age-dependent EOM rates between the settings (model 1a), we used the total daily number of EOM events as the response and the observation time during which orangutans were visible, divided by six and then log-transformed as an offset term (the mean follow duration was roughly six hours; dividing by six was done to ease model convergence;^115^). To avoid zero-inflation and overdispersion issues, we chose a model with a zero-inflated negative binomial error structure. In the zero-inflation part, we included the fixed effects of species, age, and age squared. The model comprised random slopes of age and age squared within individual, but we had to drop parameters for the correlations among random intercepts and slopes as a model including them did not converge (for final full model structure and sample sizes see SI, section C, Table S4a, b).

To compare age-dependent differences in EOM duration between the settings (model 1b), we used a linear mixed effects model (LMM) fitted with Gaussian error distribution and identity link. To achieve roughly normally distributed and homogeneous residuals (assessed by visual inspection of a qq-plot and residuals plotted against fitted values) we log-transformed (base e) the response (EOM duration).

#### EOM diversity – actions, body parts, and objects used

To investigate differences in the age-dependent development in EOM diversity (model 2a-2d), we used GLMMs with the number of different exploratory actions used during EOM (exploratory actions that included all object types (model 2a), exploratory actions during EOM events that involve only natural objects occurring at both settings (model 2b), the number of body parts used during EOM (model 2c), and whether or not multiple objects were explored during an event (i.e. one object versus more than one object, coded as 0 and 1; model 2d) as a response variable. For models 2a – 2d, the response variable was the number of unique elements per EOM event.

Since the response variable in models 2a – 2c did not comprise zeros, we fitted these models with a zero-truncated negative binomial error distribution and log link function. All four models comprised random slopes of age and age squared within individual. Model 2b also included the object as a random intercepts effect because it aimed at comparing EOM action diversity within the same object between the two settings. Initially, we also included parameters for the correlations among random intercepts and slopes. However, in each model we either had one or several absolute correlation parameters being close to one (which is indicative of them being unidentifiable^116^ or the model did not converge when we included them. We hence report results for models not comprising such correlation parameters. For the final full model structure of models 2a – 2d and the respective sample sizes see SI, section C, Table S4.

#### Age of first occurrence of specific object EOM

To model the cumulative repertoire of exploratory behaviors as a function of age (model 3) we used a specifically tailored model. The data analyzed with this model consisted of the data aggregated such that they were a series of cumulative repertoire sizes and the ages at which these were reached (e.g., 12 unique behaviors at an age of 1.62 years, 28 behaviors at an age of 1.76 years, etc.) separately for wild and zoo-housed individuals but pooled across all individuals of a given setting). To these data we then fitted an exponential function (for detailed information see SI, section D3) . We assumed R to be Poisson distributed given the fitted model (for sample sizes see SI, section C, Table S4a,b).

#### General considerations

We fitted all models in R (version 4.3.1 or higher;^117^), using the function glmmTMB of the equally named package (version 1.1.8; (Brooks et al., 2017) for models 1a, 2a, 2b, and 2c; the function lmer of the package lme4 (version 1.1–34;^118^) for model 1b and the function glmer of the same package for model 2d. Before fitting the models, we log- (base e) and then z-transformed age to a mean of zero and a standard deviation of one to ease model convergence. In the case of models 1a and 2a-d, we determined the significance of individual fixed effects of the final full model by dropping them from the model and comparing the resulting simplified model with the more complex one using a likelihood ratio test (R function drop1). For model 1b, we determined the significance of individual fixed effects of the final full model by means of the Satterthwaite approximation to degrees of freedom (function lmer of the package lmerTest; version 3.1–3;^119^; see also^120^). We estimated 95% confidence intervals of model estimates and fitted values by means of parametric bootstraps (N = 1000 bootstraps; function simulate of the package glmmTMB or function bootMer of the package lme4). For information on model stability, collinearity and overdispersion checks, see SI, section A3.

For models 1b-2d, we fitted equivalent models comprising only data collected when individuals were less than 16 years old. This was done because fine-grained effects during this period of development can be overwritten by overall trends across all ages. The details and results of these models are shown in the SI, section C and D.

We report information on median and 1^st^ and 3^rd^ quartile throughout the manuscript like this: median [1^st^ quartile, 3^rd^ quartile].

## Supplementary Information

Supplementary Information 1.

Supplementary Video 1.

Supplementary Information 2.

## Data Availability

The data is available as a supplementary file.
