# Supplementary material for: Wild and zoo-housed orangutans differ in how they explore objects
Source: Sci Rep. 2025 Apr 30;15:14853. doi: 10.1038/s41598-025-97926-z (PMC12044031; doi:10.1038/s41598-025-97926-z)
Supplement: Supplementary file 3 — Supplementary Information 2. [file 41598_2025_97926_MOESM3_ESM.docx]

**Supplementary information**

**Title:** Wild and zoo-housed orangutans differ in how they explore objects

**Authors:** Isabelle B. Laumer, Shubhangi Kansal, Anais van Cauwenberghe, Tri Rahmaeti, Tatang Mitra Setia, Roger Mundry, Daniel Haun, Caroline Schuppli

1. **Information on individual orangutans**

**Information on date of birth in wild orangutans**

For most of the wild focal individuals, the exact date of birth was unknown. We estimated the ages of our focal individuals using different approaches depending on their age-sex class at first encounter. For immatures, we used physical characteristics as well as the date their mother was last seen without the infant (i.e., before birth). In adult females, we used their total number of offspring (dependent immatures and genetically confirmed offspring) to estimate their birth date. For unflanged males, depending on their physical appearance, we assigned a standard date of birth dating fifteen to twenty years back, and for flanged males thirty years prior to the date at which they were first seen in the study area. In zoo-housed orangutans the exact birth date was usually known.

**Table S1a** Setting, site, name, age class, sex, date of birth of all participating orangutans.

| **setting** | **site** | **name** | **age class** | **sex** | **date of birth** |
| --- | --- | --- | --- | --- | --- |
| wild | Suaq | Albin | Infant | Male | 7/1/06 |
| wild | Suaq | Alice | Mother | Female | 7/1/60 |
| wild | Suaq | Amor | Infant | Male | 1/1/15 |
| wild | Suaq | Balu | Unfl.Male | Male | 6/27/92 |
| wild | Suaq | Bob | Unfl.Male | Male | 8/30/99 |
| wild | Suaq | Butterfly | Fl.Male | Male | 2/22/86 |
| wild | Suaq | Caesar | Unfl.Male | Male | 4/13/02 |
| wild | Suaq | Chick | Mother | Female | 7/1/75 |
| wild | Suaq | Chindy | Ad.Female | Female | 1/1/03 |
| wild | Suaq | Chuck | Infant | Male | 7/1/07 |
| wild | Suaq | Cinnamon | Juvenile | Female | 4/1/12 |
| wild | Suaq | Cissy | Mother | Female | 1/1/65 |
| wild | Suaq | Dalia | Juvenile | Female | 10/1/12 |
| wild | Suaq | Derek | Unfl.Male | Male | 2/19/94 |
| wild | Suaq | Dian | Fl.Male | Male | 2/9/78 |
| wild | Suaq | Diddy | Infant | Male | 12/1/05 |
| wild | Suaq | Dodi | Ad.Female | Female | 7/1/90 |
| wild | Suaq | Eddy | Fl.Male | Male | 2/9/78 |
| wild | Suaq | Eden | Infant | Female | 11/1/14 |
| wild | Suaq | Ellie | Mother | Female | 3/1/99 |
| wild | Suaq | Fez | Unfl.Male | Male | 9/30/93 |
| wild | Suaq | Filip | Unfl.Male | Male | 9/24/93 |
| wild | Suaq | Frankie | Juvenile | Male | 8/1/12 |
| wild | Suaq | Fredy | Infant | Male | 6/1/05 |
| wild | Suaq | Friska | Mother | Female | 1/1/43 |
| wild | Suaq | Gura | Unfl.Male | Male | 11/12/92 |
| wild | Suaq | Inky | Infant | Male | 7/1/03 |
| wild | Suaq | Islo | Fl.Male | Male | 9/21/77 |
| wild | Suaq | Kiki | Unfl.Male | Male | 8/13/87 |
| wild | Suaq | Kombek | Fl.Male | Male | 10/17/78 |
| wild | Suaq | Kumango | Fl.Male | Male | 4/13/88 |
| wild | Suaq | Leo | Unfl.Male | Male | 11/4/88 |
| wild | Suaq | Lilly | Mother | Female | 3/1/01 |
| wild | Suaq | Lisa | Mother | Female | 7/1/87 |
| wild | Suaq | Lois | Juvenile | Male | 8/1/10 |
| wild | Suaq | Luther | Infant | Male | 3/1/16 |
| wild | Suaq | Milo | Unfl.Male | Male | 11/24/87 |
| wild | Suaq | Nibla | Unfl.Male | Male | 8/19/92 |
| wild | Suaq | Nuk | Infant | Male | 1/1/06 |
| wild | Suaq | Otto | Fl.Male | Male | 2/7/77 |
| wild | Suaq | Pankris | Unfl.Male | Male | 8/19/87 |
| wild | Suaq | Pepito | Juvenile | Male | 1/1/13 |
| wild | Suaq | Pluto | Fl.Male | Male | 5/2/83 |
| wild | Suaq | Predator | Fl.Male | Male | 3/6/79 |
| wild | Suaq | Raffi | Mother | Female | 1/1/73 |
| wild | Suaq | Rakus | Fl.Male | Male | 3/19/89 |
| wild | Suaq | Rendang | Infant | Male | 7/15/13 |
| wild | Suaq | Robert | Unfl.Male | Male | 6/26/93 |
| wild | Suaq | Ronaldo | Infant | Male | 1/1/06 |
| wild | Suaq | Sambal | Unfl.Male | Male | 11/16/93 |
| wild | Suaq | Sarabi | Mother | Female | 1/1/85 |
| wild | Suaq | Sem | Unfl.Male | Male | 10/16/99 |
| wild | Suaq | Shane | Unfl.Male | Male | 6/5/93 |
| wild | Suaq | Shera | Juvenile | Female | 2/1/98 |
| wild | Suaq | Simba | Infant | Male | 3/1/13 |
| wild | Suaq | Smeagol | Unfl.Male | Male | 2/5/91 |
| wild | Suaq | Sumbing | Fl.Male | Male | 3/11/78 |
| wild | Suaq | Tina | Mother | Female | 1/1/98 |
| wild | Suaq | Titan | Fl.Male | Male | 6/1/83 |
| wild | Suaq | Tornado | Infant | Male | 7/1/14 |
| wild | Suaq | Trident | Mother | Female | 7/1/06 |
| wild | Suaq | Xenix | Unfl.Male | Male | 11/27/77 |
| wild | Suaq | Yoyo | Fl.Male | Male | 8/23/91 |
| wild | Suaq | Yulia | Juvenile | Female | 1/1/07 |
| wild | Suaq | Zackey | Unfl.Male | Male | 2/8/94 |
| zoo | Basel | Budi | Fl.Male | Male | 6/14/04 |
| zoo | Basel | Ketawa | Juvenile | Female | 3/3/13 |
| zoo | Basel | Maia | Mother | Female | 12/7/07 |
| zoo | Basel | Ombak | Infant | Male | 3/4/17 |
| zoo | Basel | Padma | Infant | Female | 8/7/18 |
| zoo | Basel | Revital | Mother | Female | 6/14/00 |
| zoo | Dresden | Daisy | Mother | Female | 3/20/91 |
| zoo | Dresden | Dalai | Juvenile | Male | 7/22/15 |
| zoo | Dresden | Djaka | Ad.Female | Female | 6/1/96 |
| zoo | Dresden | Djudi | Ad.Female | Female | 6/1/73 |
| zoo | Dresden | Toni | Fl.Male | Male | 12/13/91 |
| zoo | Leipzig | Lursa | Infant | Female | 8/21/21 |
| zoo | Leipzig | Padana | Ad.Female | Female | 11/18/97 |
| zoo | Leipzig | Raja | Ad.Female | Female | 9/26/03 |
| zoo | Leipzig | Sari | Juvenile | Female | 8/5/17 |
| zoo | Zurich | Cahaya | Mother | Female | 9/7/02 |
| zoo | Zurich | Djarius | Fl.Male | Male | 11/7/94 |
| zoo | Zurich | Hadiah | Unfl.Male | Male | 12/16/07 |
| zoo | Zurich | Malou | Juvenile | Male | 4/6/12 |
| zoo | Zurich | Mimpi | Juvenile | Female | 8/18/12 |
| zoo | Zurich | Pandai | Juvenile | Female | 6/12/15 |
| zoo | Zurich | Riang | Juvenile | Female | 6/12/17 |
| zoo | Zurich | Timor | Mother | Female | 11/23/75 |
| zoo | Zurich | Utu | Infant | Female | 2/4/20 |

**Table S1b** Enclosure size and enrichment scheme of the participating zoos.

| **Zoo** | **Enclosure size** | | **Examples of provided enrichment** | **Enrichment schedule** |
| --- | --- | --- | --- | --- |
|  | Indoor (m2) | outdoor (m2) |  |  |
| Leipzig Zoo | 319 | 1680 | nesting material (e.g. wood wool) | daily |
|  |  |  | enrichment material (e.g. paper, cardboard, balls, tubes) | most objects daily/some objects several times a week |
| Zurich Zoo | 86 | 50 | nesting material (e.g. wood wool, burlap sack) | daily |
|  |  |  | enrichment material (e.g. paper, paper bags, cardboard, branches, bottles, water tub, balls, tubes, burlab sacks) | most objects daily/some objects several times a week |
| Dresden Zoo | 108 | 270 | nesting material (e.g. wood wool, leaves, twigs) | daily |
|  |  |  | enrichment material (e.g. cardboard boxes, tubes, burlab sack, leaves, twigs, empty canister, water tub, clothes and fabric) | most objects daily/some objects several times a week |
| Basel Zoo | 274 | Enclosure 1: 486 | nesting material (e.g. wood wool, burlap sack) | daily |
|  |  | Enclosure 2: 440 | enrichment material (e.g. bucket, barrels, sticks, cups, bottles, clothes) | most objects daily/some objects several times a week |

For a complete list of objects provided at the zoos see Table S2b below.

**A2) Interobserver reliability**

Data were collected by 25 experienced observers. To be included in the analysis of the frequency of exploration behavior, new observers had passed an 85% inter-observer reliability test on occurrence rates of exploration behavior with an experienced observer, assessed via the 2min scan data. Data on the details of the exploration events (e.g., the duration of the events, exploratory actions, object type, and body parts used) were included after the observer went through training during which these features were discussed with an experienced observer team for each observed exploration event.

**A3) General considerations: Model stability, collinearity and overdispersion checks**

We determined model stability of models 1-2d by dropping individuals from the data set, one at a time, fitting the respective full model to each of the subsets, and then comparing the range of model estimates obtained with those of the model fitted to the full data set. All models were stable in that the estimates were consistent in their direction. To rule out collinearity issues we determined Variance Inflation Factors (VIF; (1)) of models 1-2d via the vif function of the car package (version 3.1-2; (2)) fitted to models lacking the interactions and age squared. None of the models indicated collinearity issues (maximal VIF measured across all models: 1.084, see SI, section C, Table S4). We checked models 1-2d for overdispersion, except model 1b for which this is not applicable; for all zero-truncated models we used Ben Bolker's overdispersion function (https://bbolker.github.io/mixedmodels-misc/glmmFAQ.html#overdispersion), for all other models we used a function written by RM) and detected no dispersion issues (maximum dispersion parameter = 1.489, see SI, section C, Table S4).

1. **Definitions of behavioral actions and information on bodyparts used**

**Table S2a** Detailed list of all explorative manipulations observed in this study and their definitions.

| **Exploratory behavior** | **Definitions** |
| --- | --- |
| balancing | Adjust or reposition an object on the body or another object or the ground |
| bending | Shaping an object into a curve or angle |
| biting | Cutting an object in the mouth with teeth |
| blowing | Blowing air towards an object or surface with protruded lips |
| breaking apart | Separating an object into two pieces (Snapping, not pulling apart) |
| carrying around | Transporting an object (directed movement) |
| carrying object in object | Transporting an object inside another object (directed movement) |
| catching | Reaching for an airborne object (or trying to) catch or grasp it while not changing location |
| chasing | Physically following a moving object |
| chewing | Repeatedly biting on an object that is inside the mouth |
| collecting | Moving several objects close to each other |
| covering body | Placing an object on the body to fully or mostly cover themselves |
| covering object | Placing an object over another object to partially or fully cover it (usually paper, burlap sack etc.) |
| digging | Moving body parts inside a substrate or object while breaking it up |
| dipping | Placing an object into a liquid and immediately removing it (if longer it's soaking); |
| dragging | Pulling an object over a substrate (usually the ground) without letting go of it |
| dropping | Making an object fall by letting it go of intentionally |
| filling | Insert (or facilitate insertion of) soft, loose, or liquid material into another object until full or almost full |
| fist fishing | Hitting with the fist onto a substrate on which there are insects or other objects (trying to smash them) |
| flossing | Moving an object back and forth between adjacent teeth |
| folding | Bend or rearrange an object (usually a blanket, paper, etc.) into a flatter or more compact shape |
| gnawing | Repeatedly biting on an object that is not inside the mouth |
| hand scooping | Collecting a soft, loose, or liquid material with a body part in a fluid motion |
| hanging over | Putting an object over a structure |
| harping | Moving the fingertips in a waving motion over an object or substrate as if playing the harp |
| hitting with body part | Forcefully striking a body part against an object |
| hitting with object | Forcefully striking an object/substrate with an object |
| holding object on protruded lips | Balancing object on top of upper protruded lip, while looking at it |
| holding object on top of upper lip | Balancing object on top of upper lip, while looking at it |
| hugging | Holding the body closely or tightly around an object |
| humping | Moving ones pelvic region/ hips in a forward and back motion on an object |
| intertwining | Twist and twine two objects or one long object together |
| jumping on | Purposefully pushing themselves off a surface and then on the object |
| kissing | Bring an object to the mouth or the mouth to an object and touching it with lips |
| knocking | Gently and repeatedly hitting knuckles onto a surface/object |
| knocking over | Strike or collide an object so as to cause them to fall to the ground. |
| leaning object against object/substrate | Position an object so that it balances against another object or substrate |
| licking | Passing tongue over object |
| licking body part after touch | Passing tongue over body part after touching an object with the same body part |
| lifting | Lifting up an object and then replacing it where it came from |
| lip picking | Picking an object with a protruded lip |
| looking through | Looking fixedly through an object |
| making a ball | Moving several objects together and squeezing them from several sides into a ball-like shape |
| making hole | Using force to make a hole into a substrate/object |
| masturbating | Stroking or rubbing an object on genitals |
| mixing | Combine or put together multiple objects to form one substance or mass |
| moving in/on an object | Whilst inside, covered by or on top of an object, the focal is moving or turning over and over with or on an object |
| nibbling | Gently repeatedly biting into an object with the mouth while removing small pieces |
| organizing around body | Intentionally arranging objects around themselves |
| peeing in/on object | Deliberately urinating in or on an object |
| petting | Gently stroking over an object |
| picking at | Repeatedly pulling at a small object |
| poking | Jab finger into object or substrate |
| pouring | Cause a liquid or object to flow from a container |
| pulling | Exerting force on object to cause movement towards actor |
| pulling through body part | Putting mouth or other body part around an object and then pulling the object through the mouth/ other body part |
| pulling through substrate/object | Pulling an object through a gap in a substrate, between two objects, or a space in an object |
| pushing | Exerting force on an object to cause movement away from actor |
| pushing through | Pushing an object through a gap in a substrate, between two objects, or a space in an object |
| putting lips inside an object | Pursing the lips and positioning an object to fit around them |
| putting on body part | Placing an object on a body part (distinct from covering body) |
| putting on conspecific | Placing an object on a conspecific |
| putting on head | Placing an object onto own head |
| putting one object into another | Placing one object wholly or partially inside another object |
| reaching | Reaching for an object that is out of immediate distance or failing to touch an object (usually in young infants) |
| reaching into | Sticking body part (hand arm foot head) into a substrate or object (e.g. tree hole or big piece of dead wood) |
| reaching through | Reaching through a gap/hole with a body part |
| repositioning object | Deliberately putting an object in a new position (for example: from horizontal to vertical) |
| ripping off | Tearing or pulling an object off of a substrate or other object |
| rolling object | Moving an object by turning it on a surface without lifting |
| rotating | Moving or rotating an object in a circular motion (distinct from rolling) |
| rubbing body part over object/substrate | Moving body part (except genitals - see masturbation) over an object or substrate while applying pressure |
| rubbing object against body | Moving an object against a body part repeatedly while applying pressure (different from sliding which is a singular motion) |
| rubbing object over object/substrate | Moving an object against an object or substrate repeatedly while applying pressure (different from sliding which is a singular motion) |
| scooping | Using an object as a cup |
| scraping off | Removing small parts of the object with a dragging or pulling motion across the object's surface |
| scratching | Moving fingernails over an object while applying pressure |
| shaking | Rapidly moving a detached object up and down or from side to side |
| slapping | Gently hitting an object with the palm of the hand |
| sliding apart | Pulling apart two joined objects by applying opposing force in a sliding motion (object: window cleaner) |
| sliding over surface | Pulling an object over another object or substrate in a sliding motion. |
| smashing | Hitting an object with force against a surface/ that is laying on a surface. |
| smelling | Sniffing an object |
| sniffing body part after touch | Sniffing at a body part after touching an object with the same body part |
| soaking | Holding an object in liquid allowing it to absorb the liquid |
| spitting into | Ejecting an object or water from the mouth so that it lands in another object |
| spitting out | Forcefully ejecting object from mouth making it fly away |
| squeezing | Applying force on an object from two directions to compress it |
| stacking | Arrange several objects in a vertical pile |
| stretching | Making an object longer or wider without breaking it |
| stripping off; peeling | Removing covering from an object (e.g. bark from a twig) |
| stuffing | Using force to position an object partially or fully inside an empty space |
| sucking | Drawing at an object with the mouth in a sucking motion |
| swinging object | Moving a suspended object back and forth or from side to side |
| swinging on object | Hanging from an object (that the OU placed there before) and using momentum or pushing off a surface to swing on it |
| taking apart | Deconstructing composite objects (e.g. Nests) by separating the parts |
| taking in and out of mouth | Repeatedly taking an object in and out of mouth |
| taking object out of object | Removing an object from another object/substrate |
| tearing apart | Pulling an object into two pieces by applying force |
| throwing | Propel an object with force through the air |
| throwing object onto own body | Propel an object vertically through the air so that it lands on own body |
| tilting | Holding an object and rotating it in the hands in the air in front of them without turning it all the way around |
| tool use attempt | Using a tool (here and below: a tool can be any object) to try to reach or interact with another object but failing to succeed with the tool |
| tool use collect | Using a tool to collect/gather objects together/closer |
| tool use dig | Using tool to dig |
| tool use other | Using a tool with a novel or undefined technique |
| tool use picking at | Repeatedly pulling at/removing a small object by the use of a tool |
| tool use poke | Insert a tool (mostly sticks) into explored object/ poking explored object with a tool (mostly sticks) |
| tool use pull | Pulling at an object or substrate utilizing a tool |
| tool use reach | Reaching with a tool for an object or substrate that is out of immediate distance |
| tool use rotating | Moving or rotating an object by the use of a tool in a circular motion |
| tool use scrape | Dragging or pulling a tool across the surface of an object |
| tool use stir | Moving or rotating an object by the use of a tool in a circular motion |
| tool use touch | Gently and deliberately touching an object/substrate by the use of a tool |
| tool use wave | Waving an object around by the use of a stick (object suspended on the stick) |
| touching | Deliberately touching an object with hands or feet, thereby looking at it intently, but not performing any other actions |
| transferring | Repeatedly handing an object from one body part to another |
| turning around | Turning objects around in hands and/ or feet |
| turning in mouth | Turning an object around inside the mouth without chewing on it |
| twirling | Spinning an object quickly and lightly around causing it to rotate (like a propeller) |
| twisting | Attempt to distort the shape of an object by curling it |
| waving around | Moving an object in hand or foot through the air slowly |
| winding around body | Wrapping a long object around another body part with twisting movements |
| winding around object | Wrapping a long object around another object with twisting movements |
| wiping | Gently moving object over object or substrate |

**Table S2b** Objects used during exploration in zoos, the wild, and objects used in both zoo and the wild. For the analysis 2b, we included exploration events exclusively including the following objects: “leaf and dead leaf”, “twig, stick and branch”, “wood”, “tree trunk”, “tree fork”, “bark” and, “bird nest”.

| **objects zoo** | **objects wild** | **Natural objects used in both zoo and wild** |
| --- | --- | --- |
| artificial branch |  |  |
| artificial mound |  |  |
| artificial tree |  |  |
| attached ball |  |  |
| attached tube |  |  |
| bag |  |  |
| ball |  |  |
| ball attached to rope |  |  |
| ball puzzle box |  |  |
| bamboo |  |  |
| barrell |  |  |
| bark | bark | included |
| barrel |  |  |
| bench |  |  |
|  | bird |  |
| bird nest | bird nest | included |
| bolt |  |  |
| bottle |  |  |
| branch | branch | included |
|  | branch break-off point |  |
|  | branch knob |  |
|  | bud |  |
| bucket |  |  |
| burlap sack |  |  |
| bush |  |  |
|  | cambium |  |
| cardboard |  |  |
| cardboard box |  |  |
| ceiling |  |  |
| chain |  |  |
| clothes |  |  |
| clothes keeper |  |  |
| cup |  |  |
|  | dead branch |  |
|  | dead epiphyte |  |
|  | dead flower |  |
|  | dead liana |  |
|  | dead pitcherplant |  |
| dead grass |  |  |
| dead leaf | dead leaf | included |
| dead tree | dead tree |  |
| dead treetop |  |  |
|  | dead tree fork |  |
|  | dead tree trunk |  |
|  | dead twig |  |
|  | dead vegetation |  |
|  | dead wood |  |
| detached rope |  |  |
| detached tube |  |  |
| door |  |  |
| door frame |  |  |
| door lock |  |  |
| doorstep |  |  |
|  | epiphyte |  |
| eggshell |  |  |
| enclosure attachment |  |  |
| experimental apparatus |  |  |
| fabric |  |  |
| fabric sheet |  |  |
| feather |  |  |
| feces | feces |  |
| fence |  |  |
| fence pole |  |  |
| fire hose |  |  |
| fire hose braid |  |  |
| floor |  |  |
| flour |  |  |
| flower | flower |  |
| food pellets |  |  |
| fountain |  |  |
| fruit | fruit |  |
| grass |  |  |
| hair other | hair other |  |
| body part other |  |  |
| hammock |  |  |
| hanging puzzle box |  |  |
| hinge |  |  |
|  | hollow tree |  |
| hole |  |  |
| hole in ground |  |  |
| hose |  |  |
| ice |  |  |
| insect | insect |  |
|  | insect nest |  |
|  | insect street |  |
| jug |  |  |
| leaf | leaf | included |
| liana | liana |  |
| lock |  |  |
| log with holes |  |  |
|  | moss |  |
|  | mushroom |  |
| metal plate |  |  |
| mucus |  |  |
| orangutan nest | orangutan nest |  |
|  | old orangutan nest |  |
| own body part | own body part |  |
| own hair | own hair |  |
|  | pitcherplant |  |
|  | pith |  |
| padlock |  |  |
| paper |  |  |
| paper bag |  |  |
| paper roll |  |  |
| paper sheet |  |  |
| pillar |  |  |
| pillar hole |  |  |
| pillowcase |  |  |
| plastic bottle |  |  |
| plastic bottle cap |  |  |
| plastic label |  |  |
| plastic object |  |  |
| plastic piece |  |  |
| plastic toy |  |  |
| plate |  |  |
| platform |  |  |
| pole |  |  |
| puzzle box |  |  |
| puzzle disc |  |  |
| rock |  |  |
| rod |  |  |
| roof |  |  |
| root | root |  |
| rope |  |  |
|  | sap |  |
| salt stone |  |  |
| sawdust |  |  |
| screw |  |  |
| seed | seed |  |
| sleeping box |  |  |
| sliding latch |  |  |
| snow |  |  |
| soapy water |  |  |
| soil |  |  |
| spider web |  |  |
| spit |  |  |
| stem | stem |  |
| stick | stick | included |
| sticker |  |  |
| stone |  |  |
| straw |  |  |
| swing |  |  |
| tape |  |  |
| tea |  |  |
| trash |  |  |
|  | tree |  |
|  | tree crack |  |
| tree fork | tree fork | included |
| tree hole | tree hole |  |
| tree knob | tree knob |  |
| tree trunk | tree trunk | included |
| tube |  |  |
| twig | twig | included |
|  | umbrella |  |
| urine |  |  |
| vegetation | vegetation |  |
| vertical net |  |  |
| vomit |  |  |
| wall |  |  |
| water | water |  |
| wheel |  |  |
| window |  |  |
| window cleaner |  |  |
| wire |  |  |
| wood | wood | included |
| wooden structure |  |  |

**Table S3** Detailed list of all body parts involved in explorative manipulations observed in this study in zoos and the wild.

| **body parts zoo** | **body parts wild** | **can include use of** |
| --- | --- | --- |
| arm |  |  |
| back |  |  |
| belly |  |  |
| entire body | entire body |  |
| butt |  |  |
| elbow |  |  |
| eyes | eyes |  |
| face | face |  |
| feet | feet |  |
| hands | hands | right hand, left hand, both hands, fingers, fist, and wrist |
| head | head |  |
| mouth | mouth | lips, teeth, and tongue |
| neck |  |  |
| nose | nose |  |
| shoulder | shoulder |  |

1. **Final model structure and sample sizes**

**Table S4a** Final model structure for each model.

| **age (in years)** | **model number** | **model** | **final full model structure** |
| --- | --- | --- | --- |
| all | 1a | Exploration rate | Number of exploration events ~ (age + age squared)*zoo_wild + offset(log(visible observation time/6)) + (1+age+age squared)\|\|name of individual), ziformula=~age + age squared + zoo_wild, family=nbinom2 |
| all | 1b | Duration of object exploration | Duration ~ (age + age squared)*zoo_wild+ (1 + age + age squared\|\|name of individual) + (1\|follow number in subject), REML = F |
| all | 2a | Exploration diversity: manipulations | Number of manipulations ~ (age + age squared)*zoo_wild + (1 + age + age squared\|\|name of individual) + (1\|follow number in subject), family=truncated_nbinom2 |
| all | 2b | Exploration diversity: manipulations involving objects available in both settings | Number of manipulations with objects occurring at both settings ~ (age + age squared)*zoo_wild + (1 + age + age squared\|\|name of individual) + (1\|follow number in subject)+ (1\|object), family=truncated_poisson |
| all | 2c | Exploration diversity: body parts | Number of body parts ~ (age + age squared)* zoo_wild + (1 + age + age squared\|\|name of individual) + (1\|follow number in subject), family=truncated_poisson |
| all | 2d | Exploration diversity: objects | Binary response variable multiple objects ~ (age + age squared)*zoo_wild + (1 + age + age squared\|\|name of individual) + (1\|follow number in subject), family=binomial, control=glmerControl(optimizer="nloptwrap", optCtrl=list(maxfun=100000)) |
| 0-16 | 1b | Duration of object exploration | Duration ~ (age + age squared)*zoo_wild + (1 + age + age squared\|name of individual) + (1\|follow number in subject), REML = F |
| 0-16 | 2a | Exploration diversity: manipulations | Number of manipulations ~ (age + age squared)*zoo_wild + (1 + age + age squared\|\|name of individual) + (1\|follow number in subject), family=truncated_nbinom2 |
| 0-16 | 2b | Exploration diversity: manipulations involving objects available in both settings | Number of manipulations with objects occurring at both settings ~ (age + age squared)*zoo_wild + (1 + age + age squared\|\|name of individual) + (1\|follow number in subject)+ (1\|object), family=truncated_poisson |
| 0-16 | 2c | Exploration diversity: body parts | Number of body parts ~ (age + age squared)*zoo_wild + (1 + age + age squared\|\|name of individual) + (1\|follow number in subject), family=truncated_poisson |
| 0-16 | 2d | Exploration diversity: objects | Binary response variable multiple objects ~ (age + age squared)*zoo_wild + (1 + age + age squared\|\|name of individual) + (1\|follow number in subject), family=binomial, control=glmerControl(optimizer="nloptwrap", optCtrl=list(maxfun=100000)) |
|  |  |  |  |

**Table S4b** Sample sizes, overdispersion and vif values for each model.

| **Age (in years)** | **Model number** | **Zoo: N**  **Observations** | **Zoo: N**  **individuals** | **Wild: N observations** | **Wild: N**  **individuals** | **N**  **observations total** | **N**  **‘follow number in subject’** | **N**  **individuals total** | **Overdispersion (ratio)** | **vif (log-age, setting)** |
| --- | --- | --- | --- | --- | --- | --- | --- | --- | --- | --- |
| all | 1a | 142 | 24 | 650 | 65 | 792 | NA | 89 | 1.489 | 1.001 |
| all | 1b | 5830 | 24 | 4983 | 22 | 10813 | 381 | 46 | NA | 1.067 |
| all | 2a | 6473 | 24 | 4790 | 25 | 11263 | 409 | 49 | 0.586 | 1.069 |
| all | 2b | 1085 | 21 | 2560 | 18 | 3645 | 327 | 39 | 0.562 | 1.086 |
| all | 2c | 6454 | 24 | 5480 | 27 | 11934 | 445 | 51 | 0.329 | 1.030 |
| all | 2d | 6624 | 24 | 5862 | 27 | 12486 | 458 | 51 | 0.782 | 1.062 |
| 0-16 | 1b | 5309 | 13 | 4897 | 15 | 10206 | 297 | 28 | NA | 1.042 |
| 0-16 | 2a | 5856 | 13 | 4769 | 18 | 10625 | 356 | 31 | 0.595 | 1.084 |
| 0-16 | 2b | 979 | 13 | 2558 | 16 | 3537 | 301 | 29 | 0.554 | 1.032 |
| 0-16 | 2c | 5832 | 13 | 5391 | 18 | 11223 | 359 | 31 | 0.325 | 1.024 |
| 0-16 | 2d | 5989 | 13 | 5788 | 19 | 11777 | 378 | 32 | 0.777 | 1.067 |
|  |  |  |  |  |  |  |  |  |  |  |

1. **Statistical results**
2. **Developmental trajectory and duration of object exploration (model 1a)**

**1a) Developmental trajectory of object exploration (model 1a)**

**Table S5** Results of the full model (model 1a) with exploration rate being the response.

| **term** | **Estimate** | **SE** | **CLlower** | **CLupper** | **LRT** | **df** | **p** | **min** | **max** |
| --- | --- | --- | --- | --- | --- | --- | --- | --- | --- |
| **intercept** | 0.586 | 0.190 | 0.250 | 0.894 |  |  |  | 0.486 | 0.776 |
| **age** | -2.744 | 0.334 | -3.385 | -2.112 |  |  |  | -2.880 | -2.562 |
| **age squared** | -1.211 | 0.280 | -1.801 | -0.678 |  |  |  | -1.408 | -1.030 |
| **setting** | 2.891 | 0.267 | 2.393 | 3.412 |  |  |  | 2.748 | 2.991 |
| **age:setting** | 1.447 | 0.490 | 0.472 | 2.346 |  |  |  | 1.303 | 1.609 |
| **age squared:setting** | 1.215 | 0.479 | 0.278 | 2.198 | 7.410 | 1 | 0.006 | 1.071 | 1.353 |
| **intercept (zero inflation)** | -1.276 | 0.232 | -1.937 | -0.780 |  |  |  | -1.478 | -1.049 |
| **age (zero inflation)** | 0.646 | 0.348 | -1.026 | 1.446 |  |  |  | 0.457 | 0.833 |
| **age squared (zero inflation)** | -0.111 | 0.208 | -1.549 | 0.340 | 0.297 | 1 | 0.586 | -0.220 | -0.072 |
| **setting (zero inflation)** | -1.883 | 0.571 | -3.757 | -0.903 | 15.183 | 1 | <0.001 | -2.208 | -1.677 |

indicated are estimates, together with their standard errors, 95% confidence limits, significance test, and the range of estimates obtained when excluding individuals one at a time; age was log- and then z-transformed, mean and standard variation of log-age were 2.242 and 1.092, respectively; setting was dummy coded with wild being the reference level

**1b) Duration of object exploration events (model 1b)**

**1b.1) All ages (model 1b)**

**Table S6** Results of the full model (model 1b) with log exploration duration being the response and when considering observations made at all ages.

| **term** | **Estimate** | **SE** | **CLlower** | **CLupper** | **t value** | **df** | **p** | **min** | **max** |
| --- | --- | --- | --- | --- | --- | --- | --- | --- | --- |
| **intercept** | 3.518 | 0.057 | 3.403 | 3.632 |  |  |  | 3.465 | 3.585 |
| **age** | -0.050 | 0.061 | -0.169 | 0.072 |  |  |  | -0.088 | 0.001 |
| **age squared** | 0.016 | 0.031 | -0.050 | 0.076 |  |  |  | -0.039 | 0.034 |
| **setting** | -0.096 | 0.089 | -0.270 | 0.079 |  |  |  | -0.168 | -0.040 |
| **age:setting** | 0.206 | 0.101 | -0.010 | 0.392 | 1.729 | 20 | 0.099 | 0.145 | 0.276 |
| **age squared:setting** | 0.019 | 0.052 | -0.088 | 0.124 | 0.283 | 18 | 0.780 | -0.014 | 0.075 |

indicated are estimates, together with their standard errors, 95% confidence limits, significance tests, and the range of estimates obtained when excluding individuals one at a time; age was log- and then z-transformed, mean and standard variation of log-age were 1.400 and 0.831, respectively; setting was dummy coded with wild being the reference level

**1b.2) Immature orangutans (model 1b)**

The exploration duration of immature orangutans was not significantly different between zoo and wild animals (LRT: χ2 = 0.939, df = 3, P = 0.816; Table S8; median duration _zoo_: 25 [10, 70], median duration _wild_: 30 [15, 60]).

**Table S7** Results of the full model (model 1b) with log exploration duration being the response and when considering only observations made below age 16.

| **term** | **Estimate** | **SE** | **CLlower** | **CLupper** | **t value** | **df** | **p** | **min** | **max** |
| --- | --- | --- | --- | --- | --- | --- | --- | --- | --- |
| **intercept** | 3.495 | 0.078 | 3.324 | 3.662 |  |  |  | 3.430 | 3.542 |
| **age** | 0.060 | 0.105 | -0.162 | 0.281 |  |  |  | -0.161 | 0.130 |
| **age squared** | 0.022 | 0.076 | -0.142 | 0.186 |  |  |  | -0.019 | 0.097 |
| **setting** | -0.067 | 0.124 | -0.317 | 0.216 |  |  |  | -0.157 | 0.035 |
| **age:setting** | 0.072 | 0.148 | -0.256 | 0.370 | 0.454 | 18 | 0.656 | -0.017 | 0.293 |
| **age squared:setting** | -0.013 | 0.115 | -0.272 | 0.234 | -0.071 | 14 | 0.945 | -0.120 | 0.028 |

indicated are estimates, together with their standard errors, 95% confidence limits, significance tests, and the range of estimates obtained when excluding individuals one at a time; age was log- and then z-transformed, mean and standard variation of log-age were 1.286 and 0.703, respectively; setting was dummy coded with wild being the reference level

**2) Variability of exploratory behavior (model 2a)**

**2a.1) All ages (model 2a)**

**Table S8** Results of the full model (model 2a) for the number of exploratory actions when considering individuals of all ages.

| **term** | **Estimate** | **SE** | **CLlower** | **CLupper** | **LRT** | **df** | **p** | **min** | **max** |
| --- | --- | --- | --- | --- | --- | --- | --- | --- | --- |
| **intercept** | 0.050 | 0.073 | -0.093 | 0.185 |  |  |  | -0.035 | 0.152 |
| **age** | 0.132 | 0.059 | 0.003 | 0.247 |  |  |  | 0.097 | 0.202 |
| **age squared** | -0.005 | 0.032 | -0.069 | 0.052 |  |  |  | -0.026 | 0.013 |
| **setting** | 0.424 | 0.100 | 0.231 | 0.633 |  |  |  | 0.343 | 0.489 |
| **age:setting** | 0.058 | 0.101 | -0.127 | 0.271 |  |  |  | -0.048 | 0.217 |
| **age squared:setting** | -0.058 | 0.054 | -0.150 | 0.043 | 1.251 | 1 | 0.263 | -0.117 | -0.020 |

indicated are estimates, together with their standard errors, 95% confidence limits, significance tests, and the range of estimates obtained when excluding individuals one at a time; age was log- and then z-transformed, mean and standard variation of log-age where 1.449 and 0.819, respectively; setting was dummy coded with wild being the reference level.

**Table S9** Results of the reduced model (model 2a) without the non-significant interaction between age squared and setting) for the number of exploratory actions when considering individuals of all ages.

| **term** | **Estimate** | **SE** | **CLlower** | **CLupper** | **LRT** | **df** | **p** |
| --- | --- | --- | --- | --- | --- | --- | --- |
| **intercept** | 0.077 | 0.068 | -0.064 | 0.202 |  |  |  |
| **age** | 0.133 | 0.057 | 0.009 | 0.244 |  |  |  |
| **setting** | 0.383 | 0.092 | 0.198 | 0.571 |  |  |  |
| **age squared** | -0.029 | 0.023 | -0.084 | 0.015 | 1.525 | 1 | 0.217 |
| **age:setting** | 0.013 | 0.087 | -0.152 | 0.197 | 0.023 | 1 | 0.879 |

indicated are estimates, together with their standard errors, 95% confidence limits, significance tests, and the range of estimates obtained when excluding individuals one at a time; age was log- and then z-transformed, mean and standard variation of log-age where 1.449 and 0.819, respectively; setting was dummy coded with wild being the reference level.

**Table S10** Results of the reduced model (model 2a) without the non-significant effect of age squared) for the number of exploratory actions when considering individuals of all ages.

| **term** | **Estimate** | **SE** | **CLlower** | **CLupper** | **LRT** | **df** | **p** |
| --- | --- | --- | --- | --- | --- | --- | --- |
| **intercept** | 0.045 | 0.064 | -0.086 | 0.168 |  |  |  |
| **age** | 0.132 | 0.059 | 0.014 | 0.246 |  |  |  |
| **setting** | 0.401 | 0.092 | 0.212 | 0.578 |  |  |  |
| **age:setting** | -0.019 | 0.086 | -0.187 | 0.147 | 0.049 | 1 | 0.825 |

indicated are estimates, together with their standard errors, 95% confidence limits, significance tests, and the range of estimates obtained when excluding individuals one at a time; age was log- and then z-transformed, mean and standard variation of log-age where 1.449 and 0.819, respectively; setting was dummy coded with wild being the reference level.

**Table S11** Results of the final model (model 2a; including only main effects) for the number of exploratory actions when considering individuals of all ages.

| **term** | **Estimate** | **SE** | **CLlower** | **CLupper** | **LRT** | **df** | **p** |
| --- | --- | --- | --- | --- | --- | --- | --- |
| **intercept** | 0.046 | 0.064 | -0.077 | 0.162 |  |  |  |
| **age** | 0.123 | 0.044 | 0.035 | 0.208 | 7.140 | 1 | 0.008 |
| **setting** | 0.395 | 0.088 | 0.227 | 0.576 | 15.620 | 1 | <0.001 |

indicated are estimates, together with their standard errors, 95% confidence limits, significance tests, and the range of estimates obtained when excluding individuals one at a time; age was log- and then z-transformed, mean and standard variation of log-age where 1.449 and 0.819, respectively; setting was dummy coded with wild being the reference level.

**2a.2) Immature orangutans (model 2a)**

In immature orangutans, the number of exploratory actions was significantly different between zoo-housed and wild animals (LRT: χ2 = 16.440, df = 3, P < 0.001). However, the interaction between setting and age squared was not significant in this analysis (Table S12). After removal of this non-significant interaction, we also did not find a significant effect of age squared (Table S13) which indicated linear age trajectories and led us to remove age squared from the model. In the resulting model, also the interaction between age and setting was not significant (Table S14), and we thus removed it from the model. The final model revealed significant effects of setting (χ2 = 13.472, df = 1, P < 0.001) and a marginally non-significant effect of age (χ2 = 3.706, df = 1, P = 0.054; Table S15), whereby the number of exploratory actions per event increased with age in a similar fashion in the wild and in zoos, but zoo-housed orangutans performed significantly more exploratory actions per event (Figure S1; median number of manipulations _zoo_: 2 [1, 3], median number of manipulations _wild_: 2 [1, 2]).

**Figure S1** Number of exploratory actions per event as a function of age in wild (purple) and zoo-housed orangutans (yellow) below 16 years of age (model 2a). Dashed lines and shaded areas depict the fitted model and its 95% confidence limits. Dots depict the average response per a given age, whereby their area corresponds to the number of observations per age (range: 1 to 273).

**Table S12** Results of the full model (model 2a) with number of exploratory actions being the response and when considering only observations made below 16 years.

| **term** | **Estimate** | **SE** | **CLlower** | **CLupper** | **LRT** | **df** | **p** | **min** | **max** |
| --- | --- | --- | --- | --- | --- | --- | --- | --- | --- |
| **intercept** | 0.011 | 0.079 | -0.141 | 0.155 |  |  |  | -0.087 | 0.123 |
| **age** | 0.098 | 0.079 | -0.052 | 0.257 |  |  |  | 0.053 | 0.175 |
| **age squared** | 0.024 | 0.037 | -0.056 | 0.098 |  |  |  | -0.007 | 0.050 |
| **setting** | 0.447 | 0.111 | 0.240 | 0.668 |  |  |  | 0.361 | 0.528 |
| **age:setting** | 0.102 | 0.133 | -0.164 | 0.361 | 0.621 | 1 | 0.431 | -0.030 | 0.268 |
| **age squared:setting** | -0.109 | 0.070 | -0.252 | 0.030 | 2.540 | 1 | 0.111 | -0.204 | -0.075 |

indicated are estimates, together with their standard errors, 95% confidence limits, significance tests, and the range of estimates obtained when excluding individuals one at a time; age was log- and then z-transformed, mean and standard variation of log-age where 1.338 and 0. 0.702, respectively; setting was dummy coded with wild being the reference level.

**Table S13** Results of the reduced model (model 2a; without the non-significant interaction between age squared and setting) for the number of exploratory actions when considering

only observations made below 16 years.

| **term** | **Estimate** | **SE** | **CLlower** | **CLupper** | **LRT** | **df** | **p** |
| --- | --- | --- | --- | --- | --- | --- | --- |
| **intercept** | 0.042 | 0.077 | -0.099 | 0.190 |  |  |  |
| **age** | 0.088 | 0.076 | -0.071 | 0.228 |  |  |  |
| **setting** | 0.390 | 0.106 | 0.175 | 0.603 |  |  |  |
| **age squared** | -0.008 | 0.031 | -0.073 | 0.052 | 0.061 | 1 | 0.805 |
| **age:setting** | 0.086 | 0.128 | -0.146 | 0.326 | 0.460 | 1 | 0.498 |

indicated are estimates, together with their standard errors, 95% confidence limits, significance tests, and the range of estimates obtained when excluding individuals one at a time; age was log- and then z-transformed, mean and standard variation of log-age where 1.338 and 0. 0.702, respectively; setting was dummy coded with wild being the reference level.

**Table S14** Results of the reduced model (model 2a; without the non-significant effect of age squared) for the number of exploratory actions when considering only observations made below 16 years.

| **term** | **Estimate** | **SE** | **CLlower** | **CLupper** | **LRT** | **df** | **p** |
| --- | --- | --- | --- | --- | --- | --- | --- |
| **intercept** | 0.033 | 0.070 | -0.117 | 0.171 |  |  |  |
| **age** | 0.090 | 0.076 | -0.077 | 0.246 |  |  |  |
| **setting** | 0.395 | 0.105 | 0.175 | 0.597 |  |  |  |
| **age:setting** | 0.083 | 0.129 | -0.159 | 0.364 | 0.426 | 1 | 0.514 |

indicated are estimates, together with their standard errors, confidence limits, and the range of estimates when excluding individuals one at a time; age was log- and then z-transformed, mean and standard variation of log-age where 1.338 and 0. 0.702, respectively; setting was dummy coded with being the reference level.

**Table S15** Results of the final model (model 2a; including only main effects) for the number of exploratory actions when considering only observations made below 16 years.

| **term** | **Estimate** | **SE** | **CLlower** | **CLupper** | **LRT** | **df** | **p** |
| --- | --- | --- | --- | --- | --- | --- | --- |
| **intercept** | 0.034 | 0.069 | -0.102 | 0.165 |  |  |  |
| **age** | 0.119 | 0.061 | 0.003 | 0.226 | 3.706 | 1 | 0.054 |
| **setting** | 0.415 | 0.098 | 0.227 | 0.613 | 13.472 | 1 | <0.001 |

indicated are estimates, together with their standard errors, confidence limits, significance tests, and the range of estimates obtained when excluding individuals one at a time; age was log- and then z-transformed, mean and standard variation of log-age where 1.338 and 0. 0.702, respectively; setting was dummy coded with wild being the reference level.

**2b) Variability of exploratory behavior of natural objects (model 2b)**

**2b.1) All ages (model 2b)**

**Table S16** Results of the full model **(model 2b)** with number of exploratory actions with natural items occurring at both settings being the response and when considering only observations made at all ages.

| **term** | **Estimate** | **SE** | **CLlower** | **CLupper** | **LRT** | **df** | **p** | **min** | **max** |
| --- | --- | --- | --- | --- | --- | --- | --- | --- | --- |
| **intercept** | 0.053 | 0.099 | -0.130 | 0.241 |  |  |  | 0.014 | 0.133 |
| **age** | 0.169 | 0.061 | 0.046 | 0.285 |  |  |  | 0.116 | 0.223 |
| **age squared** | -0.030 | 0.037 | -0.116 | 0.033 |  |  |  | -0.071 | -0.014 |
| **setting** | 0.315 | 0.114 | 0.091 | 0.541 |  |  |  | 0.238 | 0.370 |
| **age:setting** | -0.115 | 0.102 | -0.299 | 0.101 | 1.232 | 1 | 0.267 | -0.182 | -0.063 |
| **age squared:setting** | 0.036 | 0.054 | -0.068 | 0.149 | 0.426 | 1 | 0.514 | 0.009 | 0.076 |

indicated are estimates, together with their standard errors, 95% confidence limits, significance tests, and the range of estimates obtained when excluding individuals one at a time; age was log- and then z-transformed, mean and standard variation of log-age where 1.273 and 0. 0.810, respectively; setting was dummy coded with wild being the reference level.

**Table S17** Results of the first reduced model (model 2b; lacking the interaction between age squared and setting) with number of exploratory actions with natural objects being the response and when considering observations made at all ages.

| **term** | **Estimate** | **SE** | **CLlower** | **CLupper** | **LRT** | **df** | **p** |
| --- | --- | --- | --- | --- | --- | --- | --- |
| **intercept** | 0.035 | 0.095 | -0.140 | 0.220 |  |  |  |
| **age** | 0.171 | 0.062 | 0.043 | 0.287 |  |  |  |
| **setting** | 0.345 | 0.106 | 0.156 | 0.564 |  |  |  |
| **age squared** | -0.013 | 0.027 | -0.073 | 0.039 | 0.262 | 1 | 0.609 |
| **age:setting** | -0.094 | 0.098 | -0.287 | 0.121 | 0.886 | 1 | 0.346 |

indicated are estimates, together with their standard errors, 95% confidence limits, significance tests, and the range of estimates obtained when excluding individuals one at a time; age was log- and then z-transformed, mean and standard variation of log-age where 1.273 and 0. 0.810, respectively; setting was dummy coded with wild being the reference level.

**Table S18** Results of the second reduced model (model 2b; lacking age squared) with number of exploratory actions with natural objects being the response and when considering observations made at all ages.

| **term** | **Estimate** | **SE** | **CLlower** | **CLupper** | **LRT** | **df** | **p** |
| --- | --- | --- | --- | --- | --- | --- | --- |
| **intercept** | 0.022 | 0.092 | -0.171 | 0.187 |  |  |  |
| **age** | 0.174 | 0.062 | 0.044 | 0.291 |  |  |  |
| **setting** | 0.350 | 0.106 | 0.146 | 0.553 |  |  |  |
| **age:setting** | -0.114 | 0.089 | -0.290 | 0.075 | 1.577 | 1 | 0.209 |

indicated are estimates, together with their standard errors, 95% confidence limits, significance tests, and the range of estimates obtained when excluding individuals one at a time; age was log- and then z-transformed, mean and standard variation of log-age where 1.273 and 0. 0.810, respectively; setting was dummy coded with wild being the reference level.

**Table S19** Results of the third reduced model (model 2b; lacking the interaction between age and setting) with number of exploratory actions with natural objects being the response and when considering observations made at all ages.

| **term** | **Estimate** | **SE** | **CLlower** | **CLupper** | **LRT** | **df** | **p** |
| --- | --- | --- | --- | --- | --- | --- | --- |
| **intercept** | 0.022 | 0.092 | -0.176 | 0.188 |  |  |  |
| **age** | 0.118 | 0.047 | 0.024 | 0.208 | 5.299 | 1 | 0.021 |
| **setting** | 0.311 | 0.101 | 0.110 | 0.522 | 8.462 | 1 | 0.004 |

indicated are estimates, together with their standard errors, 95% confidence limits, significance tests, and the range of estimates obtained when excluding individuals one at a time; age was log- and then z-transformed, mean and standard variation of log-age where 1.273 and 0. 0.810, respectively; setting was dummy coded with wild being the reference level.

**2b.2) Immatures (model 2b)**

In immature orangutans, the number of exploratory actions with natural objects that were present in both settings was significantly different between zoo-housed and wild animals (LRT: χ2 = 10.199, df = 3, P = 0.017). The two-way interaction between age squared and setting was not significant (Table S20), thus we removed it from the model. In the resulting model, we did not find a significant effect of age squared (Table S21), and hence we removed it from the model, too. The resulting model did not reveal a significant effect of the interaction between age and setting, and thus we removed it from the model (Table S22). The final model revealed a significant effect of setting (χ2 = 7.619, df = 1, P = 0.006, Table S23) but not of age. Plotting the model and the data revealed that zoo-housed orangutans showed a higher number of exploratory actions with natural items available in both settings than wild orangutans (Figure S2; median number of exploratory actions _zoo_ =2 [1, 3], median number of exploratory actions _wild_ = 2 [1, 2]).

**Figure S2)** Number of exploratory actions with natural objects available in both settings per event as a function of age in wild (purple) and zoo-housed orangutans (yellow) below 16 years of age (model 2b). Dashed lines and shaded areas depict the fitted model and its 95% confidence limits. Dots depict the average response per a given age and setting, whereby their area corresponds to the number of exploration events per age and setting (range: 1 to 70).

**Table S20** Results of the full model (model 2b) with number of exploratory actions with natural objects being the response and when considering only observations made below 16 years.

| **term** | **Estimate** | **SE** | **CLlower** | **CLupper** | **LRT** | **df** | **p** | **min** | **max** |
| --- | --- | --- | --- | --- | --- | --- | --- | --- | --- |
| **intercept** | 0.027 | 0.106 | -0.184 | 0.233 |  |  |  | -0.029 | 0.110 |
| **age** | 0.154 | 0.067 | 0.012 | 0.285 |  |  |  | 0.101 | 0.218 |
| **age squared** | -0.021 | 0.041 | -0.101 | 0.064 |  |  |  | -0.070 | 0.023 |
| **setting** | 0.425 | 0.135 | 0.142 | 0.700 |  |  |  | 0.310 | 0.517 |
| **age:setting** | -0.168 | 0.118 | -0.402 | 0.074 | 1.925 | 1 | 0.165 | -0.248 | -0.030 |
| **age squared:setting** | -0.073 | 0.081 | -0.249 | 0.080 | 0.843 | 1 | 0.358 | -0.132 | -0.035 |

Indicated are estimates, together with their standard errors, 95% confidence limits, significance tests, and the range of estimates obtained when excluding individuals one at a time; age was log- and then z-transformed, mean and standard variation of log-age where 1.211 and 0.739, respectively; setting was dummy coded with wildbeing the reference level.

**Table S21** Results of the first reduced model (model 2b; lacking the interaction between age squared and setting) with number of exploratory actions with natural objects being the response and when considering observations only made below the age of 16.

| **term** | **Estimate** | **SE** | **CLlower** | **CLupper** | **LRT** | **df** | **p** |
| --- | --- | --- | --- | --- | --- | --- | --- |
| **intercept** | 0.051 | 0.102 | -0.145 | 0.259 |  |  |  |
| **age** | 0.147 | 0.065 | 0.003 | 0.279 |  |  |  |
| **setting** | 0.361 | 0.114 | 0.128 | 0.579 |  |  |  |
| **age squared** | -0.041 | 0.035 | -0.116 | 0.025 | 1.394 | 1 | 0.238 |
| **age:setting** | -0.161 | 0.114 | -0.390 | 0.077 | 1.937 | 1 | 0.164 |

Indicated are estimates, together with their standard errors, 95% confidence limits, significance tests, and the range of estimates obtained when excluding individuals one at a time; age was log- and then z-transformed, mean and standard variation of log-age where 1.211 and 0.739, respectively; setting was dummy coded with wild being the reference level.

**Table S22** Results of the second reduced model (model 2b; lacking age squared) with number of exploratory actions with natural objects being the response and when considering observations only made below the age of 16.

| **term** | **Estimate** | **SE** | **CLlower** | **CLupper** | **LRT** | **df** | **p** |
| --- | --- | --- | --- | --- | --- | --- | --- |
| **intercept** | 0.005 | 0.096 | -0.182 | 0.202 |  |  |  |
| **age** | 0.161 | 0.065 | 0.029 | 0.296 |  |  |  |
| **setting** | 0.374 | 0.116 | 0.152 | 0.604 |  |  |  |
| **age:setting** | -0.178 | 0.115 | -0.413 | 0.060 | 2.312 | 1 | 0.128 |

Indicated are estimates, together with their standard errors, 95% confidence limits, significance tests, and the range of estimates obtained when excluding individuals one at a time; age was log- and then z-transformed, mean and standard variation of log-age where 1.211 and 0.739, respectively; setting was dummy coded with wild being the reference level.

**Table S23** Results of the third reduced model (model 2b; lacking the interaction between age and setting) with number of exploratory actions with natural objects being the response and when considering observations only made below the age of 16.

| **term** | **Estimate** | **SE** | **CLlower** | **CLupper** | **LRT** | **df** | **p** |
| --- | --- | --- | --- | --- | --- | --- | --- |
| **intercept** | 0.007 | 0.096 | -0.192 | 0.199 |  |  |  |
| **age** | 0.100 | 0.057 | -0.015 | 0.213 | 2.699 | 1 | 0.100 |
| **setting** | 0.324 | 0.110 | 0.114 | 0.564 | 7.619 | 1 | 0.006 |

Indicated are estimates, together with their standard errors, 95% confidence limits, significance tests, and the range of estimates obtained when excluding individuals one at a time; age was log- and then z-transformed, mean and standard variation of log-age where 1.211 and 0.739, respectively; setting was dummy coded with wild being the reference level.

**2c) Variability of body parts used during object exploration (model 2c)**

**2c.1. All ages (model 2c)**

**Table S24** Results of the full model (model 2c) with number of body parts being the response and when considering all ages.

| **term** | **Estimate** | **SE** | **CLlower** | **CLupper** | **LRT** | **df** | **p** | **min** | **max** |
| --- | --- | --- | --- | --- | --- | --- | --- | --- | --- |
| **intercept** | -0.380 | 0.147 | -0.656 | -0.087 |  |  |  | -0.449 | -0.183 |
| **age** | 0.053 | 0.103 | -0.149 | 0.253 |  |  |  | 0.007 | 0.130 |
| **age squared** | -0.143 | 0.045 | -0.244 | -0.055 |  |  |  | -0.160 | -0.078 |
| **setting** | 0.065 | 0.213 | -0.364 | 0.447 |  |  |  | -0.058 | 0.155 |
| **age:setting** | 0.421 | 0.172 | 0.106 | 0.764 | 6.885 | 1 | 0.009 | 0.274 | 0.558 |
| **age squared:setting** | 0.015 | 0.075 | -0.163 | 0.169 | 0.042 | 1 | 0.838 | -0.029 | 0.044 |

indicated are estimates, together with their standard errors, 95% confidence limits, significance tests, and the range of estimates obtained when excluding individuals one at a time; age was log- and then z-transformed, mean and standard variation of log-age where 1.395 and 0.865, respectively; setting was dummy coded with wild being the reference level.

**Table S25** Results of the reduced model (model 2c; lacking the interaction between age squared and setting) with number of body parts being the response and when considering all ages.

| **term** | **Estimate** | **SE** | **CLlower** | **CLupper** | **LRT** | **df** | **p** |
| --- | --- | --- | --- | --- | --- | --- | --- |
| **intercept** | -0.387 | 0.143 | -0.677 | -0.111 |  |  |  |
| **age** | 0.050 | 0.103 | -0.151 | 0.248 |  |  |  |
| **setting** | 0.077 | 0.204 | -0.332 | 0.470 |  |  |  |
| **age squared** | -0.138 | 0.037 | -0.217 | -0.069 | 11.304 | 1 | 0.001 |
| **age:setting** | 0.435 | 0.160 | 0.147 | 0.745 | 8.600 | 1 | 0.003 |

indicated are estimates, together with their standard errors, 95% confidence limits, significance tests, and the range of estimates obtained when excluding individuals one at a time; age was log- and then z-transformed, mean and standard variation of log-age where 1.395 and 0.865, respectively; setting was dummy coded with wild being the reference level.

**2c.2) Immature orangutans (model 2c)**

For immature orangutans, the model with the number of body parts involved in exploration events revealed a significantly full-null model comparison (LRT: χ2 = 12.829, df = 3, P = 0.005). The interaction between age squared and location was not significant in this model (Table S26). After its removal, the final model revealed a significant effect of age squared (χ2 = 9.710, df = 1, P = 0.002; SI, Table S27) and the interaction of age and setting (χ2 = 11.040, df = 1, P = 0.001; SI, Table S27), whereby in wild animals the number of body parts peaked at the age of two to three years of age and then decreased, while in zoo-housed orangutans it clearly increased with age (see Figure S3; median number of body parts_zoo_ = 1 [1, 2], median number of body parts_wild_ = 1 [1,2]).

**Figure S3** Number of body parts per event as a function of age in wild (purple) and zoo-housed orangutans (yellow) below 16 years of age (model 2c). Dashed lines and shaded areas depict the fitted model and its 95% confidence limits. Dots depict the average response per a given age, whereby their area corresponds to the number of observations per age (range: 1 to 276).

**Table S26** Results of the full model (model 2c) with number of body parts being the response and when considering only individuals below 16 years.

| **term** | **Estimate** | **SE** | **CLlower** | **CLupper** | **LRT** | **df** | **p** | **min** | **max** |
| --- | --- | --- | --- | --- | --- | --- | --- | --- | --- |
| **intercept** | -0.314 | 0.161 | -0.639 | -0.001 |  |  |  | -0.372 | -0.099 |
| **age** | -0.190 | 0.150 | -0.460 | 0.097 |  |  |  | -0.260 | -0.144 |
| **age squared** | -0.203 | 0.050 | -0.307 | -0.100 |  |  |  | -0.233 | -0.188 |
| **setting** | -0.101 | 0.234 | -0.546 | 0.375 |  |  |  | -0.259 | -0.019 |
| **age:setting** | 0.724 | 0.227 | 0.290 | 1.188 | 10.268 | 1 | 0.001 | 0.556 | 0.860 |
| **age squared:setting** | 0.135 | 0.107 | -0.135 | 0.346 | 1.539 | 1 | 0.215 | 0.086 | 0.245 |

indicated are estimates, together with their standard errors, 95% confidence limits, significance tests, and the range of estimates obtained when excluding individuals one at a time; age was log- and then z-transformed, mean and standard variation of log-age were 1.273 and 0. 0.737, respectively; setting was dummy coded with wild being the reference level.

**Table S27** Results of the final reduced model (model 2c; lacking the interaction age squared and setting) with number of body parts being the response and when considering only individuals below 16 years.

| **term** | **Estimate** | **SE** | **CLlower** | **CLupper** | **LRT** | **df** | **p** |
| --- | --- | --- | --- | --- | --- | --- | --- |
| **intercept** | -0.326 | 0.154 | -0.620 | -0.016 |  |  |  |
| **age** | -0.210 | 0.156 | -0.490 | 0.078 |  |  |  |
| **setting** | -0.060 | 0.221 | -0.484 | 0.384 |  |  |  |
| **age squared** | -0.176 | 0.044 | -0.264 | -0.083 | 9.710 | 1 | 0.002 |
| **age:setting** | 0.776 | 0.237 | 0.323 | 1.275 | 11.040 | 1 | 0.001 |

indicated are estimates, together with their standard errors and 95% confidence limits, significance tests; age was log- and then z-transformed, mean and standard variation of log-age were 1.273 and 0. 0.737, respectively; setting was dummy coded with wild being the reference level.

**2d) Variability of objects used and object-object interactions during object exploration (model 2d)**

**2d.1) All ages (model 2d)**

**Table S28** Results of the full model (model 2d) with multiple objects involved (yes/no) being the response and when considering observations made at all ages.

| **term** | Estimate | SE | CLlower | CLupper | LRT | df | p | min | max |
| --- | --- | --- | --- | --- | --- | --- | --- | --- | --- |
| **intercept** | -3.630 | 0.143 | -3.906 | -3.392 |  |  |  | -3.741 | -3.385 |
| **age** | 0.343 | 0.147 | 0.056 | 0.629 |  |  |  | 0.276 | 0.455 |
| **age squared** | 0.034 | 0.074 | -0.127 | 0.172 |  |  |  | -0.004 | 0.064 |
| **setting** | 1.633 | 0.218 | 1.276 | 2.049 |  |  |  | 1.425 | 1.817 |
| **age:setting** | 0.610 | 0.246 | 0.165 | 1.101 | 6.147 | 1 | 0.013 | 0.473 | 0.812 |
| **age squared:setting** | -0.338 | 0.124 | -0.591 | -0.108 | 5.924 | 1 | 0.015 | -0.432 | -0.283 |

Indicated are estimates, together with their standard errors, 95% confidence limits, significance tests, and the range of estimates obtained when excluding individuals one at a time; age was log- and then z-transformed, mean and standard variation of log-age where, 1.387 and 0.851 respectively; setting was dummy coded with wild being the reference level.

**2d.2) Immatures (model 2d)**

In immatures, the number of exploration events involving more than one object was significantly different between wild and zoo-housed orangutans (LRT: χ2 = 29.943, df = 3, P < 0.001). The interaction between age squared and setting was not significant in this model (Table S29). After removal of this non-significant interaction, we also did find a significant effect of age squared (Table S30) which led us to remove age squared from the model. The final model revealed a significant interaction between age and setting (Table S31) in that zoo-housed orangutans performed significantly more exploration events involving more than one object and the number of exploration events involving more than one object increased with age (see Figure S4).

**Figure S4** Probability of exploration events involving multiple objects per event as a function of age in wild (purple) and zoo-housed orangutans (yellow) of all ages (model 2d). Dots depict the average response per a given age, whereby their area corresponds to the number of observations per age in both figures (range: 1 to 280). Dashed lines and shaded areas in both figures depict the fitted model and its 95% confidence limits.

**Table S29** Results of the full model (model 2d) with multiple objects involved (yes/no) being the response and when considering observations only made below the age of 16.

| **term** | **Estimate** | **SE** | **CLlower** | **CLupper** | **LRT** | **df** | **p** | **min** | **max** |
| --- | --- | --- | --- | --- | --- | --- | --- | --- | --- |
| intercept | -3.676 | 0.168 | -3.996 | -3.379 |  |  |  | -3.903 | -3.516 |
| age | 0.215 | 0.201 | -0.157 | 0.584 |  |  |  | 0.108 | 0.474 |
| age squared | 0.019 | 0.117 | -0.210 | 0.217 |  |  |  | -0.105 | 0.128 |
| setting | 1.373 | 0.284 | 0.906 | 1.859 |  |  |  | 1.173 | 1.758 |
| age:setting | 0.616 | 0.306 | 0.038 | 1.272 | 3.754 | 1 | 0.053 | 0.331 | 0.731 |
| age squared: setting | -0.075 | 0.202 | -0.508 | 0.289 | 0.139 | 1 | 0.709 | -0.264 | 0.118 |

indicated are estimates, together with their standard errors, 95% confidence limits, significance tests, and the range of estimates when excluding individuals one at a time; age was log- and then z-transformed, mean and standard variation of log-age where 1.271 and 0.728, respectively; setting was dummy coded with being the reference level.

**Table S30** Results of the first reduced model (model 2d; lacking the interaction between age squared and setting) with multiple objects involved (yes/no) being the response and when considering observations only made below the age of 16.

| **term** | **Estimate** | **SE** | **CLlower** | **CLupper** | **LRT** | **df** | **p** |
| --- | --- | --- | --- | --- | --- | --- | --- |
| **intercept** | -3.656 | 0.158 | -3.945 | -3.364 |  |  |  |
| **age** | 0.201 | 0.201 | -0.160 | 0.555 |  |  |  |
| **setting** | 1.318 | 0.232 | 0.878 | 1.733 |  |  |  |
| **age squared** | 0.003 | 0.108 | -0.195 | 0.163 | 0.001 | 1 | 0.980 |
| **age: setting** | 0.614 | 0.309 | 0.037 | 1.167 | 3.655 | 1 | 0.056 |

indicated are estimates, together with their standard errors, 95% confidence limits, significance tests, and the range of estimates when excluding individuals one at a time; age was log- and then z-transformed, mean and standard variation of log-age where 1.271 and 0.728, respectively; setting was dummy coded with being the reference level.

**Table S31** Results of the second reduced model (model 2d; lacking age squared) with multiple objects involved (yes/no) being the response and when considering observations only made below the age of 16.

| **term** | **Estimate** | **SE** | **CLlower** | **CLupper** | **LRT** | **df** | **p** |
| --- | --- | --- | --- | --- | --- | --- | --- |
| **intercept** | -3.654 | 0.133 | -3.925 | -3.417 |  |  |  |
| **age** | 0.201 | 0.200 | -0.154 | 0.569 |  |  |  |
| **setting** | 1.318 | 0.232 | 0.912 | 1.739 |  |  |  |
| **age:setting** | 0.614 | 0.307 | 0.051 | 1.167 | 4.110 | 1 | 0.043 |

indicated are estimates, together with their standard errors, 95% confidence limits, significance tests, and the range of estimates when excluding individuals one at a time; age was log- and then z-transformed, mean and standard variation of log-age where 1.271 and 0.728, respectively; setting was dummy coded with being the reference level.

1. **Age of first occurrence of specific object exploration behavior**

To model the cumulative repertoire of exploratory behaviors as a function of age we used a specifically tailored model. We fitted an exponential function. The principal structure of the exponential function was R = *C* + *a* × *b*^age, where R is a sequence of cumulative repertoire sizes, age is a sequence of ages at which the cumulative repertoires had been reached, and *C*, *a*, and *b* are model parameters to be estimated, while C is the final asymptote, that is, the repertoire size approached as age increases (towards infinity). The parameter *a*, together with *C*, determines the repertoire size when age is zero. As this can reasonably be assumed to be zero, too, the function simplifies to R = *C* - *C* × *b*^age. The last parameter, *a*, determines the steepness of the trajectory. Since it seemed likely that the age-dependent accumulation of the behavioral repertoire would have different asympotes in the zoo and in the wild and also that the pace of increase would differ between settings, we modified the function to R = *C* + *Cs* × *Ds* - (*C* + *Cs × Ds*) × (*b + bs* × *Ds*)^age, where *Cs* and *bs* are two further model parameters to be estimated which estimate the magnitude of the difference between zoo and wild animals with regard to the asymptote and steepness of the trajectory, and *Ds* is a dummy variable indicating whether the respective data point is from a wild (*Ds* = 0) or zoo setting (*Ds* = 1). We fitted the model using two functions that we wrote, utilizing the R function optim with the default optimizer Nelder-Mead. Internal transformation ensured that (*C* + *Cs* × *Ds*) was positive and that (*b + bs* × *Ds*) was bound between zero and one. Furthermore, the function called optim iteratively, each time using the results of the previous call as starting parameters for the current call until the log-likelihood did not change by more than 0.001. We assumed R to be Poisson distributed given the fitted model (for sample sizes see SI, section C, Table S4a,b).

**3a) Age of first occurrence of specific object exploration**

**Table S32** Age of first occurrence of all exploratory object manipulation behaviors of wild and zoo-housed orangutans.

| **Exploratory behaviors** | **Age of 1st occurrence in zoos** | **Age of 1st occurrence in wild** |
| --- | --- | --- |
| balancing | 4.38 | NA |
| bending | 0.77 | 0.68 |
| biting | 0.62 | 0.52 |
| blowing | 5.85 | NA |
| breaking apart | 1.11 | 0.52 |
| carrying around | 0.62 | 0.68 |
| carrying object in object | 3.13 | NA |
| catching | 5.56 | 4.28 |
| chasing | NA | 4.28 |
| chewing | 0.62 | 0.52 |
| collecting | 1.34 | NA |
| covering body | 0.77 | NA |
| covering object | 2.84 | NA |
| digging | 3.97 | 0.68 |
| dipping | 3.13 | NA |
| dragging | 5.96 | NA |
| dropping | 0.62 | 0.52 |
| filling | 3.98 | NA |
| fist fishing | NA | 0.54 |
| flossing | 1.10 | NA |
| folding | 0.77 | NA |
| gnawing | 0.62 | 0.92 |
| hand scooping | 5.55 | 2.70 |
| hanging over | 5.96 | NA |
| harping | 5.85 | NA |
| hitting with body part | 0.77 | 0.68 |
| hitting with object | 5.15 | 2.84 |
| holding object on protruded lips | 5.96 | 0.92 |
| holding object on top of upper lip | 30.30 | NA |
| hugging | 1.51 | NA |
| humping | 3.98 | NA |
| intertwining | 3.97 | 4.94 |
| jumping on | 1.34 | NA |
| kissing | 0.62 | 0.54 |
| knocking | 3.79 | NA |
| knocking over | 2.51 | NA |
| leaning object against object/substrate | 5.96 | NA |
| licking | 0.62 | 0.55 |
| licking body part after touch | 1.73 | 0.93 |
| lifting | 0.62 | 6.52 |
| lip picking | 0.62 | 2.26 |
| looking through | 1.11 | NA |
| making a ball | 3.79 | 7.16 |
| making hole | 5.15 | NA |
| masturbating | 7.25 | NA |
| mixing | 4.81 | NA |
| moving in/on an object | 1.73 | 6.52 |
| nibbling | 0.62 | 0.92 |
| organizing around body | 1.51 | NA |
| peeing in/on object | 3.13 | NA |
| petting | 0.62 | 0.87 |
| picking at | 0.62 | 0.92 |
| poking | 1.11 | 0.87 |
| pouring | 3.13 | NA |
| pulling | 0.62 | 0.52 |
| pulling through body part | 1.11 | 0.89 |
| pulling through substrate/object | 1.34 | NA |
| pushing | 1.10 | 1.36 |
| pushing through | 1.34 | NA |
| putting lips inside an object | 1.11 | NA |
| putting on body part | 1.34 | NA |
| putting on conspecific | 5.85 | NA |
| putting on head | 1.11 | 1.66 |
| putting one object into another | 3.79 | 6.53 |
| reaching | 4.64 | 0.92 |
| reaching into | 1.11 | 0.92 |
| reaching through | 5.85 | NA |
| repositioning object | 1.51 | NA |
| ripping off | 0.77 | 0.53 |
| rolling object | 0.62 | 4.97 |
| rotating | 5.96 | NA |
| rubbing body part over object/substrate | 0.77 | 2.08 |
| rubbing object against body | 6.00 | 6.51 |
| rubbing object over object/substrate | 1.10 | NA |
| scooping | 3.13 | NA |
| scraping off | 1.11 | 2.29 |
| scratching | 0.77 | 0.88 |
| shaking | 0.77 | 0.53 |
| slapping | 1.11 | 0.68 |
| sliding apart | 4.64 | NA |
| sliding over surface | 3.13 | NA |
| smashing | 1.10 | NA |
| smelling | 0.62 | 0.69 |
| sniffing body part after touch | 1.51 | 0.70 |
| soaking | 3.79 | NA |
| spitting into | 1.73 | NA |
| spitting out | 1.34 | 1.59 |
| squeezing | 0.62 | NA |
| stacking | 3.97 | NA |
| stretching | 1.51 | NA |
| stripping off; peeling | 1.11 | 2.71 |
| stuffing | 4.38 | 2.29 |
| sucking | 0.62 | 0.53 |
| swinging object | 0.62 | 0.52 |
| swinging on object | 1.11 | NA |
| taking apart | 1.34 | 2.25 |
| taking in and out of mouth | 0.62 | 0.54 |
| taking object out of object | 5.85 | NA |
| tearing apart | 0.77 | 0.88 |
| throwing | 1.10 | 0.53 |
| throwing object onto own body | 5.85 | NA |
| tilting | 2.51 | NA |
| tool use attempt | 2.78 | 2.39 |
| tool use collect | 9.67 | NA |
| tool use dig | 4.81 | NA |
| tool use other | 1.34 | NA |
| tool use picking at | 5.96 | NA |
| tool use poke | 1.73 | 2.70 |
| tool use pull | 4.64 | NA |
| tool use reach | 5.26 | NA |
| tool use rotating | 6.38 | NA |
| tool use scrape | 0.77 | 2.99 |
| tool use stir | 9.98 | NA |
| tool use touch | 5.96 | NA |
| tool use wave | 9.30 | NA |
| touching | 0.62 | 0.55 |
| transferring | 1.73 | 2.41 |
| turning around | 1.10 | 0.69 |
| turning in mouth | 0.62 | 1.59 |
| twirling | 1.10 | 2.72 |
| twisting | 1.10 | NA |
| waving around | 0.62 | 0.54 |
| winding around body | 1.11 | 0.87 |
| winding around object | 2.78 | NA |
| wiping | 5.96 | NA |

**3b) Number of exploratory behaviors as a function of age of first occurrence**

**Table S33** Estimated parameters of the non-linear model used to analyze repertoire accumulation in zoo and wild orangutans.

|  | **estimate** | **SE** | **z** | **P** |
| --- | --- | --- | --- | --- |
| **c** | 4.483 | 0.12 | 37.468 | 0 |
| **b** | 1.571 | 0.188 | 8.342 | 0 |
| **cz** | 0.472 | 0.129 | 3.654 | 0 |
| **bz** | -0.044 | 0.21 | -0.21 | 0.834 |

**References:**

1. Field A. Discovering statistics using SPSS (Introducing statistical methods). SAGE Publications, London. 2005.

2. Fox J, Weisberg S. An R companion to applied regression: Sage publications; 2011.
